# Pressure‐dependent regulation of Ca^2+^ signalling in the vascular endothelium

**DOI:** 10.1113/JP271157

**Published:** 2015-12-07

**Authors:** Calum Wilson, Christopher D. Saunter, John M. Girkin, John G. McCarron

**Affiliations:** ^1^ Strathclyde Institute of Pharmacy and Biomedical SciencesUniversity of StrathclydeSIPBS Building, 161 Cathedral StreetGlasgowG4 0REUK; ^2^ Centre for Advanced Instrumentation, Biophysical Sciences Institute, Department of PhysicsDurham UniversitySouth RoadDurhamDH1 3LEUK

## Abstract

**Key points:**

- Increased pressure suppresses endothelial control of vascular tone but it remains uncertain (1) how pressure is sensed by the endothelium and (2) how the vascular response is inhibited.
- This study used a novel imaging method to study large numbers of endothelial cells in arteries that were in a physiological configuration and held at normal blood pressures.
- Increased pressure suppressed endothelial IP_3_‐mediated Ca^2+^ signals.
- Pressure modulated endothelial cell shape.
- The changes in cell shape may alter endothelial Ca^2+^ signals by modulating the diffusive environment for Ca^2+^ near IP_3_ receptors.
- Endothelial pressure‐dependent mechanosensing may occur without a requirement for a conventional molecular mechanoreceptor.

**Abstract:**

The endothelium is an interconnected network upon which haemodynamic mechanical forces act to control vascular tone and remodelling in disease. Ca^2+^ signalling is central to the endothelium's mechanotransduction and networked activity. However, challenges in imaging Ca^2+^ in large numbers of endothelial cells under conditions that preserve the intact physical configuration of pressurized arteries have limited progress in understanding how pressure‐dependent mechanical forces alter networked Ca^2+^ signalling. We developed a miniature wide‐field, gradient‐index (GRIN) optical probe designed to fit inside an intact pressurized artery that permitted Ca^2+^ signals to be imaged with subcellular resolution in a large number (∼200) of naturally connected endothelial cells at various pressures. Chemical (acetylcholine) activation triggered spatiotemporally complex, propagating inositol trisphosphate (IP_3_)‐mediated Ca^2+^ waves that originated in clusters of cells and progressed from there across the endothelium. Mechanical stimulation of the artery, by increased intraluminal pressure, flattened the endothelial cells and suppressed IP_3_‐mediated Ca^2+^ signals in all activated cells. By computationally modelling Ca^2+^ release, endothelial shape changes were shown to alter the geometry of the Ca^2+^ diffusive environment near IP_3_ receptor microdomains to limit IP_3_‐mediated Ca^2+^ signals as pressure increased. Changes in cell shape produce a geometric microdomain regulation of IP_3_‐mediated Ca^2+^ signalling to explain macroscopic pressure‐dependent, endothelial mechanosensing without the need for a conventional mechanoreceptor. The suppression of IP_3_‐mediated Ca^2+^ signalling may explain the decrease in endothelial activity as pressure increases. GRIN imaging provides a convenient method that gives access to hundreds of endothelial cells in intact arteries in physiological configuration.

## Key points

- Increased pressure suppresses endothelial control of vascular tone but it remains uncertain (1) how pressure is sensed by the endothelium and (2) how the vascular response is inhibited.
- This study used a novel imaging method to study large numbers of endothelial cells in arteries that were in a physiological configuration and held at normal blood pressures.
- Increased pressure suppressed endothelial IP_3_‐mediated Ca^2+^ signals.
- Pressure modulated endothelial cell shape.
- The changes in cell shape may alter endothelial Ca^2+^ signals by modulating the diffusive environment for Ca^2+^ near IP_3_ receptors.
- Endothelial pressure‐dependent mechanosensing may occur without a requirement for a conventional molecular mechanoreceptor.

**Abbreviations**
[Ca^2+^]_c_: cytosolic Ca^2+^ concentration
GRIN: gradient index
IP_3_: inositol trisphosphate
IP_3_R: inositol trisphosphate receptor
RyR: ryanodine receptor
TRP: transient receptor potential

## Introduction

The vascular endothelium is a one‐cell‐thick layer that directs the formation of new blood vessels (angiogenesis), prevents blood clotting, regulates vascular permeability, controls arterial tone and determines the extent of smooth muscle proliferation. The endothelium's control of each of these functions arises from the cells acting as a sensitive signal processing centre that detects and interprets multiple simultaneous messages such as those derived from mechanical stimuli (hydrostatic pressure, luminal shear stress, circumferential strain) and local and blood borne signals (autocrine, paracrine and electrical signals and neurotransmitters). Endothelial stimuli are transduced to changes in the endothelial Ca^2+^ concentration to coordinate the endothelium's control of vascular activity (Behringer & Segal, 2012; Sonkusare *et al*. 2012; Billaud *et al*. 2014). Ca^2+^ regulates the synthesis and release of various vasoactive agents such as nitric oxide, prostacyclin and endothelium‐derived hyperpolarizing factor. Through these Ca^2+^‐dependent mediators the endothelium's control of vascular contraction, permeability, cell proliferation and angiogenesis is achieved. Therefore, central to an understanding of endothelial signal processing is an appreciation of the control of Ca^2+^.

Two main sources of endothelial Ca^2+^ are recognized, the extracellular fluid and the intracellular stores of the endoplasmic reticulum (Moccia *et al*. 2012). Ca^2+^ entry from the extracellular fluid may occur via a large number of ion channels on the outside (plasma) membrane. The other main cytoplasmic Ca^2+^ concentration ([Ca^2+^]_c_) source is the internal endoplasmic reticulum store from which release proceeds mainly via the inositol 1,4,5‐trisphosphate (IP_3_) receptor (IP_3_R). While Ca^2+^ release via IP_3_R is well established the contribution of the ryanodine receptor (RyR) to the control of endothelial Ca^2+^ (if any) is less clear (Socha *et al*. 2012 *a*). The sources of Ca^2+^ are not independent: Ca^2+^ influx regulates Ca^2+^ release and Ca^2+^ release regulates Ca^2+^ influx. For example, Ca^2+^ release from the endoplasmic reticulum may alter the activity of ion channels present on the plasma membrane to regulate Ca^2+^ entry either via Ca^2+^‐gated ion channels (Strotmann *et al*. 2003) or via membrane potential changes altering passive fluxes of the ion (Behringer & Segal, 2015). Alternatively Ca^2+^ influx may alter endoplasmic reticulum Ca^2+^ content or activity of IP_3_R in a Ca^2+^‐induced Ca^2+^ release‐like process (Earley & Brayden, 2015). Thus the local change in [Ca^2+^]_c_ arising from the activity of channels in the plasma membrane or endoplasmic reticulum itself regulates the activity of ion channels to provide a feedback control of Ca^2+^ signals and modulate vascular function.

The response to mechanical stimuli involves changes in [Ca^2+^]_c_ and must be integrated with signals from other sensors to converge on a physiological response. Two major mechanical stimuli are shear stress and pressure (Falcone *et al*. 1993; Huang *et al*. 1998; Popp *et al*. 1998; Muller *et al*. 1999; Marchenko & Sage, 2000; Paniagua *et al*. 2000; Sun *et al*. 2001; Duza & Sarelius, 2004). The endothelial response to shear stress is well characterized and several types of activity may occur. Vascular smooth muscle relaxation may be evoked, cell migration induced and endothelial gene expression changed (Falcone *et al*. 1993; Muller *et al*. 1999; Shiu *et al*. 2004; Chien, 2007). The increases in endothelial Ca^2+^ changes underlying these responses may involve several types of mechanically sensitive ion channels forces such as Piezo1 (Li *et al*. 2014; Ranade *et al*. 2014), ENaC (Kusche‐Vihrog *et al*. 2014), ATP‐gated P2X4 (Yamamoto *et al*. 2006), TREK‐1 (Dedman *et al*. 2009) and various members of the TRP grouping of channels (Corey *et al*. 2004; Maroto *et al*. 2005; Spassova *et al*. 2006; Janssen *et al*. 2011). Alternatively, shear stress‐evoked activation of ion channels may be indirect and force sensed by the cytoskeleton, apical glycocalyx, mechanosensitive or membrane curvature‐sensitive protein complexes or G‐protein‐coupled receptors (Knudsen & Frangos, 1997; Thi *et al*. 2004; Tzima *et al*. 2005; Zimmerberg & Kozlov, 2006; Mederos y Schnitzler *et al*. 2008; Zhao *et al*. 2011). The diversity of responses highlights the complexity of the response to shear stress.

The response of the endothelium to pressure differs significantly from that of shear stress. Rather than being activated, a *decrease* in activity may occur as mechanical stimulation (pressure) is increased (Hishikawa *et al*. 1992; Gunduz *et al*. 2008). The decrease in endothelial activity may suppress smooth muscle relaxation (De Bruyn *et al*. 1994; Huang *et al*. 1998; Paniagua *et al*. 2000; Zhao *et al*. 2015). In healthy human volunteers, short‐term increases in arterial blood pressure cause long‐lasting inhibition of endothelium‐dependent dilatation (Jurva *et al*. 2006; Phillips *et al*. 2011). In isolated arteries, acute exposure to increases in transmural pressure may impair endothelium‐dependent relaxation (Hishikawa *et al*. 1992; Huang *et al*. 1998; Paniagua *et al*. 2000; Zhao *et al*. 2015). Relatively little is known on how pressure is sensed by the endothelium, specifically when the arteries are in their natural ‘tubular’ configuration.

The biological response of arteries to pressure depends critically on the complex, cylindrical, three‐dimensional arrangement of cells and on the interactions with other cell types, and may change with artery configuration. For example, in many intact arteries in their cylindrical configuration, myogenic contraction occurs when the artery is subjected to circumferential stretch by increases in pressure. However, this contraction does not occur in arteries stretched, with equivalent forces, on wires (Dunn *et al*. 1994). Increased mechanical stimulation by stretch, hypotonic cell swelling or shear stress activates TRPV4 channels (Strotmann *et al*. 2000; Alessandri‐Haber *et al*. 2003; Loukin *et al*. 2010), a mechanism that may contribute to flow‐induced dilatation (Kohler *et al*. 2006; Mendoza *et al*. 2010; Bubolz *et al*. 2012). However, TRPV4 channels are also deactivated by increased stretch when transmural pressure difference is increased in pressurized arteries (Bagher *et al*. 2012) in a cylindrical configuration. The cell shape and operative mechanical forces in a pressurized artery are quite different from experiments on isolated or cultured cells.

The carotid artery contributes to the control of cerebrovascular blood flow and cerebral vascular resistance (Mchedlishvili, 1986; Faraci & Heistad, 1990). The endothelium regulates carotid artery tone. Vascular relaxation is evoked by several endothelial activators including flow (shear stress), adrenonedullin and acetylcholine (Faraci *et al*. 1994; Plane *et al*. 1998; Chataigneau *et al*. 1998 *a*,*b*; Ohashi *et al*. 2005) and the endothelium may also attenuate the vascular contractile response to vasoconstrictors (Lamping & Faraci, 2001). Many of the most serious forms of cardiovascular diseases (e.g. atherosclerosis) reside in larger arteries like the carotid artery and begin with endothelial dysfunction (Deanfield *et al*. 2007). However, studying the effects of mechanical forces like pressure on endothelial function (and dysfunction) in larger arteries in a physiological configuration has been exceptionally difficult. Assessment of endothelial function in large arteries has been largely indirect. The majority of papers in the past decade involved only the measurement of endothelium‐dependent dilatation (e.g. Craig & Martin, 2012).

To study the function of endothelial cells in intact arteries, some investigations have used either wide‐field or point‐scanning fluorescence microscopes to visualize the endothelium through the wall of the artery (Bagher *et al*. 2012; Sonkusare *et al*. 2012; Tran *et al*. 2012). However, movement of the artery in pressure myograph systems is almost unavoidable and results in the vessel moving in and out of the focal plane, changing light levels and altering image quality to present a significant challenge to data analysis. Light scattering by the artery wall also reduces contrast and the curvature of the artery limits the number of cells that may be visualized within a single optical plane. To overcome each of these difficulties and allow the endothelium to be studied in intact arteries, we have developed a miniature optical probe to record Ca^2+^ signalling from *inside* pressurized arteries. The probe has a field of view of 0.5 mm diameter, which allowed a large number (∼200) of naturally connected endothelial cells to be imaged with subcellular resolution and has a high depth of field (141 μm) sufficient to maintain good focus across the highly curved, intact endothelial layer of a large artery. To handle the data from such a large number of cells a largely automated image processing routine was also developed. We show in native endothelial cells in their physiological configuration, that acetylcholine‐evoked Ca^2+^ rises originate as IP_3_‐mediated Ca^2+^ signals in particular regions of the endothelium from which they progress to other cells as Ca^2+^ waves. Detection of pressure‐dependent mechanical forces by the endothelium is integrated effortlessly into the same signalling pathway by geometric modulation of IP_3_‐evoked Ca^2+^ release brought about by changes in endothelial cell shape. We suggest the suppression of IP_3_‐mediated Ca^2+^ signals may underlie the inhibition of endothelial responses with increased pressure and, significantly, may not require a conventional mechanosensor for mechanotransduction to occur.

## Methods

### Ethical approval

All experiments employed tissue obtained from male Sprague–Dawley rats (10–12 weeks old; 250–350 g). Rats were humanely killed by overdose of pentobarbital sodium with the approval of the University of Strathclyde Local Ethical Review Panel (200 mg kg^−1^; Schedule 1 procedure; Animals (Scientific Procedures) Act 1986, UK), under UK Home Office Project and Personal Licence authority.

### Tissue preparation

The left and right common carotid arteries were exposed by blunt dissection. To prevent collapse of the artery upon removal, the rostral and caudal ends of the exposed carotid arteries were ligated with 8‐0 suture and the arteries were then rapidly excised. Arteries were then cleaned of connective tissue under a dissection microscope, and visually checked for the presence of side branches. Subsequently, arteries without side branches were mounted onto two blunted and deburred 22‐gauge cannula in a custom‐designed imaging bath using two lengths of suture thread. Blood was removed from the arteries by flushing the lumen with physiological saline solution (PSS) for 10 min (150 μl min^−1^) before the arteries were pressurized to 60 mmHg, gently heated to 37°C and allowed to equilibrate at 37^o^C for 30 min. Flow was provided by a peristaltic pump (PS‐200, Living Systems Instrumentation, At Albans City, VT, USA) connected to the proximal cannula. The endothelium of mounted arteries was then selectively loaded with a fluorescent Ca^2+^ indicator by intraluminal perfusion of PSS containing the membrane‐permeant form of Oregon Green BAPTA‐1 (20 μm, OGB‐1/AM) and 0.04% Pluronic acid. Flow was stopped for 30 min to permit sufficient loading. Arteries were continuously superfused with PSS during this time. Following removal of excess dye, the distal cannula was removed from the artery and the artery was mounted on a gradient index (GRIN) microprobe (described below) and secured with suture thread. Transmural pressure was then incrementally increased to 160 mmHg, whilst stretching the artery to remove any buckle. Following this procedure, the pressure was decreased to 60 mmHg and the artery left for a further 30 min to equilibrate. During equilibration arteries were tested for leaks (which may indicate side branches, tears in the vessel wall or insufficiently tied sutures) by switching off the feedback on the pressure servo. Arteries that showed signs of leakage, identified as a reduction in pressure whilst isolated from the servo system, which could not be stopped by re‐tying the sutures were discarded at this point.

### Micro‐endoscope GRIN imaging probe

**Figure 1:**
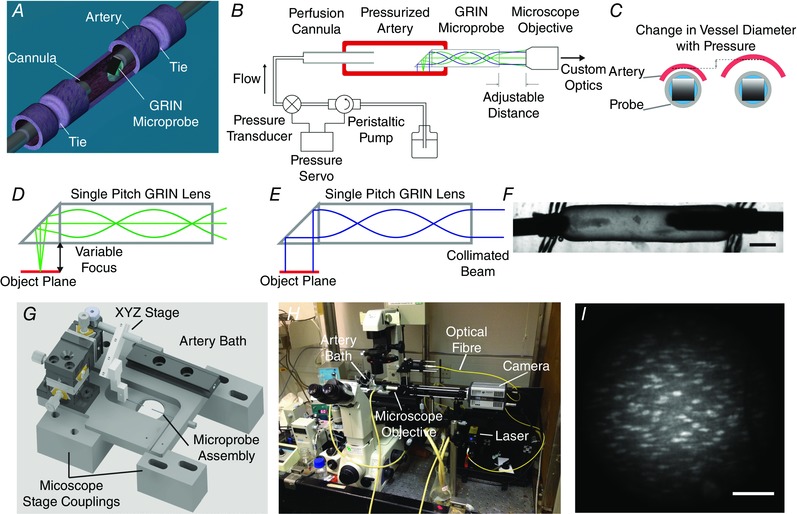
**Endothelial GRIN imaging system** *A*, cartoon showing the GRIN microprobe assembly inside a pressurized artery and endothelium. *B*, a simplified schematic diagram of the GRIN imaging system and the cannulated, pressurized artery. Focusing into the GRIN microprobe with a conventional microscope objective extended the image plane beyond the front surface of the prism and onto the endothelium. The connecting perfusion cannula (*B*) and pressure servo system permit intraluminal pressure to be controlled and the probe can be refocused if the artery position moves (*C*). *D*, schematic diagram illustrating the optical emission path through the GRIN microprobe. The GRIN lens reconjugates the image plane of a conventional microscope through the length of the cylinder. *E*, schematic diagram illustrating the optical excitation path through the GRIN microprobe. The collimated input excitation light is re‐collimated at the output of the GRIN lens. *F*, a transmission image of a cannulated artery shows the GRIN microprobe on the right‐hand side. *G*, 3‐dimensional rendering of the custom pressure arteriograph showing the position of the GRIN microprobe assembly and XYZ translation stage for positioning the artery. *H*, picture of set‐up during live imaging showing the camera and laser arrangement. *I*, fluorescence image of the endothelium visualized using the GRIN imaging system showing some activated cells (see online Supporting information, Movie S1). Note the field of view is circular due to the cross‐sectional shape of the GRIN lens. Scale bar 100 μm.

The GRIN microprobe was developed to fit inside pressurized arteries (Fig. 1 *A*) and consisted of a 0.5 mm diameter, 30.2 mm long, single pitch GRIN relay lens (SRL‐050; Nippon Sheet Glass, USA) with a 0.5 mm × 0.5 mm × 0.5 mm aluminium‐coated micro‐prism (66‐771; Edmund Optics, USA) attached to the distal surface with ultraviolet curing optical epoxy (NAO 68; Norland Products, USA). The lens was sheathed in a surgical stainless steel tube (0.71 mm outer diameter) for mechanical protection. In this single lens configuration, the GRIN rod acts to reconjugate the image plane of detection optics through the length of the cylinder (Fig. 1 *D*). By focusing a conventional microscope objective to a sufficient depth inside the GRIN rod, the image plane can be extended beyond the front surface of the prism and into tissue (Fig. 1 *C–E*). This property renders the probe suitable for the replacement of a cannula in a custom‐made pressure myograph, where the vessel must be tied to the probe to maintain pressure (Fig. 1 *B*). The focal plane of the optical system can be varied in response to diameter variations in the vessel (Fig. 1 *C*), without moving the probe, by focusing the microscope objective (Fig. 1 *B*) further into the GRIN lens (see also Flusberg *et al*. 2008; Kim *et al*. 2010).

A 0.1 NA GRIN probe was used to ensure a good depth of focus (141 μm) across the entire endothelial surface of curved artery. However, the use of a low NA GRIN relay lens imposes two opposing constraints on the transmission of light through the system. First, to maximize the delivery of excitation light through the GRIN rod, the NA of the excitation light should not be greater than the NA of the GRIN lens (Fig. 1 *D*). Second, to maximize fluorescence detection, the NA of the collection lens should be higher than that of the GRIN rod. The solution to these opposing constraints is to illuminate the proximal end of the GRIN lens with collimated light, which is re‐collimated at the output (Fig. 1 *E*) (Saunter *et al*. 2012) evenly illuminating the entire field of view whilst keeping the illumination constant despite probe refocusing.

The assembled GRIN probe was held in place in a custom‐designed arteriograph (Fig. 1 *G*) that was mounted on the microscope stage of a standard inverted microscope (Fig. 1 *H*). The fluorescence excitation and delivery system was constructed using 30 mm cage system components (Thorlabs, UK), and was also mounted onto the microscope stage via a three‐axis translation stage. Two axes of the translation stage permitted the external optical system to be coupled to the GRIN microprobe, whilst the third enabled the focus of the system to be altered. Mounting both the arteriograph and the external optical system on the stage ensured that movement of the microscope stage did not decouple the probe from the system. Fluorescence excitation was provided by a fibre‐coupled diode‐pumped solid‐state laser operating at 488 nm, which was collimated (Fig. 1 *B* and *H*) before being focused by another lens and guided to the back of a 20 × 0.5 NA infinity‐corrected microscope objective (Plan Fluor; Nikon, UK) via a dichroic mirror. Optical excitation power density, measured at the output of the GRIN probe was 1 nW μm^2^. The microscope objective and a 65 mm focal length tube lens imaged fluorescence emission, returning through the probe, through an emission filter and onto a sCMOS camera (Zyla 3‐Tap; Andor, UK) controlled by μManager (Edelstein *et al*. 2010), providing an effective pixel size of 1 μm at the object plane and permitting up to 200 endothelial cells to be imaged with subcellular resolution (see below, Optical characterization).

### Optical characterization

**Figure 2:**
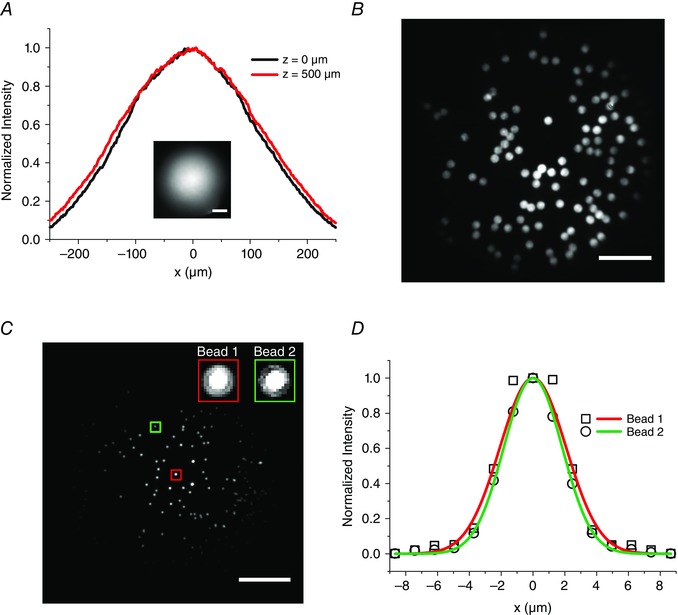
**Optical characterization of the side‐viewing GRIN imaging system** *A*, normalized intensity profiles, plotted as a function of radius (*x*) from the centre of the circular cross‐section of the GRIN lens, of images of fluorescein solution (1 μm) taken with the GRIN microendoscope as the focus was translated along the *z*‐axis. *B*, fluorescence image of 15 μm fluorescent spheres. *C*, raw image of sub‐resolution (1.0 μm) fluorescent beads obtained with the microendoscopic imaging system. Vignetting and heterogeneous fluorescence emission from the beads themselves result in apparent variation in size. By rescaling the intensity of the image (insets), beads of apparently different diameter are shown to be approximately equal in size. *D*, normalized fluorescence intensity profiles, with Gaussian curves fitted, of the two beads highlighted in *A*. From such intensity profiles, we calculated the optical resolution of our system to be 4.51 ± 0.04 μm (*n* = 6 beads). Scale bars: 100 μm.

To demonstrate the fluorescence signal detected by the microendoscope camera, we recorded images of a diffuse fluorescein solution (1 μm; Fig. 2 *A*), and of large 15.45 ± 0.04 μm (mean ± standard deviation; dimensions provided by manufacturer) diameter fluorescent beads (FS07F; Bang Laboratories Inc., USA; Fig. 2 *B*). To image fluorescein fluorescence, the bath chamber was filled with the fluorescent solution. Large‐diameter fluorescent beads were diluted in water (100× dilution in water) and left to settle on the microprobe prism surface. Figure 2 *A* shows the normalized intensity across the centre of the circular field of view (raw image of fluorescein fluorescence inset). Due to vignetting of the illumination light and of the fluorescence excitation by the cylindrically shaped GRIN lens, the efficiency drops to 50% at a distance approximately 150 μm from the centre of the probe. Translating the focal plane 500 μm along the *z*‐axis, beyond the distal prism surface resulted in a slight increase in fluorescence signal, relative to maxima. Note that due to the curvature of pressurized arteries, this intensity profile does not necessarily reflect that obtained in imaging experiments performed in intact arteries. Average optical excitation power density (1 nW μm^−2^) was calculated across the full 500 μm field of view from power measurements made at the output of the GRIN microprobe using a photodiode power sensor and power meter (200–1100 nm; S120VC and PM100A; Thorlabs, UK).

To study the resolving power of the micro‐endoscope, we imaged subresolution 1 μm fluorescent microspheres (F‐8823; Invitrogen, UK). The mean fluorescent microsphere diameter (as provided by the manufacturer) was 1.1 μm with a coefficient of variation (standard deviation/mean) of 4%. A small droplet of a fluorescent microsphere suspension (1 × 10^6^ dilution in water) was manually pipetted onto the distal prism surface. Fluorescent beads were left to settle on the distal prism surface before being brought into focus and imaged (Fig. 2 *C*). Line intensity profiles of the fluorescence emitted from individual beads were taken (Fig. 2 *D*). Gaussian fits of these line intensity profiles yielded a lateral resolution of 4.51 ± 0.04 μm (*n* = 6 beads). This measured value may be considered to be the actual resolution of our system because it is much larger than the actual size of the beads (Kim *et al*. 2010). All optical characterization experiments were performed using the same set‐up and external optics as that used for intraluminal imaging of the endothelium.

### Ca^2+^ imaging

Endothelial Ca^2+^ signals were recorded (5 Hz) from ∼200 endothelial cells in each artery by GRIN microendoscopy. The plane of focus of the GRIN imaging system was set to the endothelial layer. Arteries at resting diameter were stimulated by application of ACh (100 μm), delivered by a handheld pipette to the outside of the pressurized artery to confirm endothelial viability. Preliminary experiments established that ∼80% of the cells in the field of view responded to ACh (100 μm). Arteries in which the majority of endothelial cells did not exhibit a Ca^2+^ response to ACh were discarded. To examine the effect of increases in transmural pressure, endothelial Ca^2+^ signalling was recorded at pressures within the physiological range for the artery (60 mmHg, 110 mmHg and 160 mmHg). Following each change in pressure, the imaging system was refocused on the endothelium and arteries were left to equilibrate for 20 min. The Ca^2+^ responses evoked by ACh at various transmural pressures were studied in single experiments and expressed relative to the control response (60 mmHg). There was significant overlap in the cells imaged at each pressure, but matching individual cells was not possible. In other experiments, the extraluminal PSS was exchanged for PSS containing various pharmacological inhibitors or Ca^2+^‐free PSS and left for 20 min, before activation again with 100 μm extraluminally applied ACh. The Ca^2+^ responses evoked by ACh in arteries treated with pharmacological inhibitors or Ca^2+^‐free bath solution were studied (control and treatment) in the same artery and expressed relative to the control response. Following each acquisition period, the bath solution was immediately exchanged and the arteries were left for at least 20 min to re‐equilibrate. Smooth muscle cells were not loaded with OGB‐1/AM in any of our preparations, as indicated by an absence of fluorescence staining orthogonal to the longitudinal vessel axis.

### Signal analysis

Individual endothelial cells were segmented using a custom, semi‐automated image processing procedure. Previous successful fully automated image processing of endothelial Ca^2+^ signals has been achieved by assigning regions of interest (ROIs), of predetermined size and shape to image sequences based on subcellular activity (Francis *et al*. 2012). Here, the activity of entire cells was used to determine the shape of individual cellular ROIs. In detail, a series of images was created to illustrate the active wavefronts by generating the forward differences of the cytoplasmic Ca^2+^ concentration ([Ca^2+^]_c_) change. First, to facilitate visual inspection of endothelial Ca^2+^ signals, the active Ca^2+^ wavefronts themselves were examined by generating the forward difference of [Ca^2+^]_c_ changes (*F_t_* − *F_t_*_−1,_ obtained by sequential subtraction (SS); Bradley *et al*. 2003; McCarron *et al*. 2010). Then single images, illustrating all endothelial cells exhibiting ACh‐evoked Ca^2+^ activity, were created by taking projections of the standard deviation (STDev) of intensity of SS image stacks. Unsharp masking of standard deviation projections was used to create sharpened, background‐corrected STDev images, where ROIs encompassing individual cells could be easily obtained by intensity thresholding. ROIs corresponding to individual cell outlines were verified for each image series and erroneous ROIs were corrected manually. Cell outlines were stored as polygon descriptions within text (.txt) files for subsequent processing and analysis. Except for the creation of standard deviation projections (performed in FIJI; Schindelin *et al*. 2012) and for manually splitting joined cells, all processing was performed using batch‐processing algorithms in ImagePro Plus.

Individual fluorescence signals were extracted for each polygonal region from the raw image stacks using a custom program in the Python language. Fluorescence signals are expressed as baseline corrected values (*F*/*F*_0_), calculated by dividing the raw signals by the average value of using a user‐defined period (typically 50 frames) preceding ACh‐evoked Ca^2+^ activity. There was a significant variation in the time taken for each cell to respond to ACh. To aid analysis, individual *F*/*F*_0_ traces were aligned with respect to their peak rate of change. The alignment provides a clear illustration of total Ca^2+^ activity. Baseline values of *F*/*F*_0_, peak amplitudes and the time of peak rate of change for each signal were calculated automatically and stored as data tables within .csv files. These .csv files were then imported into Origin 9.1 for calculation of peak changes in fluorescence intensity (Δ*F*/*F*_0_), and for plotting using custom analysis scripts. For presentation, an 6‐point (1.2 s), third‐order polynomial Savitzky–Golay filter was applied within Origin; all measurements were from unsmoothed traces.

### Measurement of arterial diameter

At the end of some experiments, we recorded the diameter of arteries as pressure was increased, in 5 mmHg increments, from 0 mmHg to 200 mmHg. For videomicroscopy‐based diameter measurements, arteries were illuminated with bright field illumination, which was guided to a CCD camera (Sony XC‐77; Sony, Japan) mounted on the side‐port of the inverted microscope on which our GRIN imaging system was mounted. Images were captured from the CCD camera using Micromanager software (Edelstein *et al*. 2010), and a USB video capture device (Dazzle; Pinnacle Systems, USA) and stored on a computer for subsequent analysis. Due to light scattering by the artery wall, the luminal diameter could not be assessed. Thus, outer artery diameter was measured using the Vessel Diameter plugin for ImageJ (Fischer *et al*. 2010).

### Histological analysis

Rat carotid arteries were fixed at pressure after length adjustment (to remove buckle) at 160 mmHg. Following length adjustment, lumenal PSS was replaced with Zenker's fixative. Once the lumen was filled with fixative, the artery was sealed and the pressure was immediately raised to 60 mmHg or 160 mmHg, and the extraluminal PSS was replaced with Zenker's solution. Arteries were left to fix for at least 2 h – a time determined, in preliminarily experiments to be required to prevent a reduction in arterial dimensions upon removal of pressure. Following fixation, arteries were removed from the myography chamber, washed overnight in tap water and stored in 70% ethanol at 4°C until use. Arteries were paired for analysis (i.e. two arteries from each animal were fixed at the two different pressures (60 and 160 mmHg).

Following fixation, arteries were dehydrated in alcohol series (70% ethanol, 4 h, 4 changes; 95% ethanol, 2 h, 2 changes; 100% ethanol, 2 h, 4 changes), cleared (1:1 mixture of 100% ethanol, 1 h; Histo‐Clear (National Diagnostics, Atlanta, GA, USA), overnight, 4 changes), infiltrated and embedded (paraffin wax, 4 h). Wax blocks were cut into 5 μm thick sections and mounted onto slides. Slides were rehydrated in Histo‐Clear (10 min, 1 change), then alcohol series (100% ethanol, 10 min, 1 change; 95% ethanol, 2 min; 70% ethanol, 2 min), before being washed in distilled water. Rehydrated slides were stained with Harris's haematoxylin solution (10 min), washed with warm running tap water (10 min) then Scotts tap water (1 min), differentiated in acid alcohol (0.3%, 5 s), stained with eosin (5 min), washed with warm running tap water (10 min), then dehydrated in alcohol series (70% ethanol, 2 min; 95% ethanol, 2 min; 100% ethanol, 10 min, 1 change), cleared in Histo‐Clear (10 min, 1 change), before being mounted with Histo‐Clear. Stained artery cross‐sections were imaged using a Leica DM LB2 microscope with a Leica DFC320 camera (Leica Microsystems, UK). The thickness of endothelial cell nuclei, used as an indication of height of the endothelial cell layer, were measured using Image Pro Plus.

### Solutions and drugs

PSS consisted of (mm): NaCl (145), KCl (4.7), Mops (2.0), NaH_2_PO_4_ (1.2) glucose (5.0), ethylenediamine‐tetraacetic acid (EDTA, 0.02), sodium pyruvate (2.0), MgCl_2_ (1.17), CaCl_2_ (2.0) (pH adjusted to 7.4 with NaOH). In Ca^2+^‐free PSS, no Ca^2+^ was added, the concentration of MgCl_2_ was increased to 3.17 mm, and ethylene glycol tetraacetic acid (EGTA, 1 mm) was included.

Although a relatively high extraluminal concentration of ACh was required to activate the majority of endothelial cells, it is likely that the endothelium was not exposed to this concentration due to the presence of an adventitial barrier to diffusion. The concentration of ACh in the lumen in the present experiments was estimated to be ∼100‐fold less than the bath concentration at the time of Ca^2+^ measurement. Two experiments support this conclusion. First, in arteries surgically opened, ACh (1 μm) produced approximately equivalent responses (i.e. ∼80% cells responding) to ACh (100 μm) applied to the bath in intact artery. Secondly, the concentration of ACh was also estimated from the time course of diffusion of fluorescein across the vascular wall in the same experimental conditions as the pressurized artery. The fractional fluorescence change (relative to the final steady‐state value) at the time (10 s after addition) that ACh evoked Ca^2+^ responses was used to estimate the fraction of the ACh present in the lumen (∼1 μm). In other studies, the potency of extraluminally applied ACh was reported to be ∼1/50 of intraluminally applied ACh in the dog mesenteric artery (Toda *et al*. 1990) and 50–100 times less potent in femoral artery (Angus *et al*. 1983; Toda *et al*. 1988). Additionally bradykinin is unable to evoke relaxant responses in isolated porcine coronary arteries when applied extraluminally, independent of enzymatic degradation and luminal pressure, but is able to evoke responses when applied intraluminally (Tanko *et al*. 1999).

Drugs were all obtained from Sigma except for OGB‐1/AM and Pluronic F‐127, which were obtained from Invitrogen. All drugs were dissolved in DMSO and diluted to working concentration in PSS such that the total volume of DMSO was less than or equal to 0.1%.

Harris's haematoxylin, Scotts tap water and Eosin were obtained from and Sigma (UK). Zenker's fixative was obtained from Fisher Scientific.

### Statistics

Summarized data are expressed as means ± standard error of the mean (SEM). One‐way ANOVA (with Tukey's *post hoc* test as appropriate) was used for comparisons between groups, and biological replicate (animal) was treated as a random factor. Statistical analyses were performed using Minitab 17 (Minitab Inc., USA). A *P*‐value less that 0.05 was considered significant and *n* is number of animals.

### Model description

To understand how changes in cell geometry alter IP_3_‐evoked Ca^2+^ release, local [Ca^2+^] in the IP_3_ receptor (IP_3_R) microdomain was determined computationally in the time period encompassing ion channel opening. The cytosolic concentrations of ionic Ca^2+^, buffer and buffered Ca^2+^ are represented by *C*_Ca, cyt_, *C*_B, cyt_ and *C*_CaB, cyt_ respectively. The partial differential equation governing the concentration, *C*_s_, of a species, s, is given by:

(1) $$\frac{\partial C_{s}}{\partial t}=D_{s}\nabla^{2}C_{s}+\varphi_{s}+J_{s}$$

where *C*_s_ is the concentration, *D*_s_ is the diffusion coefficient, ϕ_s_ is the source term derived from chemical reactions and *J*_s_ is the source term resulting from trans‐membrane flux, which was taken as zero for all species except Ca^2+^, for which it comprises IP_3_R‐mediated Ca^2+^ currents.

#### Buffering

We employed the first order mass action reaction kinetic:

(2) φ Ca , cyt =−K on C Ca , cyt CB, cyt +K off C CaB , cyt

(3) φB, cyt =φ CaB , cyt =−φ Ca , cyt

where *K*_on_ and *K*_off_ are the rate constants for the buffer.

#### Trans‐membrane flux

Ca^2+^ pumps (SERCAs) and membrane (plasma and ER) leakage currents are typically continuous, low magnitude processes that function to maintain specific concentrations of Ca^2+^ in the cytosol (typically < 100 nm) and the ER (typically > 0.5 mm) in the long term (Table 1). This is in contrast to IP_3_Rs, which are reported to open for durations of between 2 ms and 20 ms, with a transient current that is far higher than those of the aforementioned long‐term processes. We therefore omitted the slow acting sources and sinks, as their effects over the brief temporal and spatial scales of an individual microdomain, with which we are concerned, are limited. The transport of Ca^2+^ from the ER to the cytosol, through an open IP_3_R, is a purely diffusive process, and is therefore driven in linear proportion to the ionic concentration gradient between the two partitions. Therefore individual IP_3_R are represented by the source term:

(4) Js=αJ0C Ca , er −C Ca , cyt C Ca , er

where *C*_Ca,er_ is the concentration of Ca^2+^ in the ER, taken as a constant, *J*_0_ is the experimentally measured maximal ion current of an isolated IP_3_R and α is a conversion factor from a current (in moles per second) to a molar concentration for the voxel to which the current is applied.

**Table 1:** **Numerical parameters used in reaction diffusion simulations**

Grid and solver			References and notes
Voxel Scale	*dx*	0.015 μm	
Solver time step	*dt*	0.120 μs	
Courant–Freidcrchs–Lewy condition [Ca^2+^]	2*Ddt dx* ^−2^ must be < 1	0.23	Evaluated for Ca^2+^ as most diffusive
Cytosolic Ca^2+^			
Equilibrium concentration	*C*_inf_	70 nm	Huang *et al*. (2000); Means *et al*. (2006); Bortolozzi *et al*. (2008)
Diffusion coefficient	*D*_Ca,cyt_	220 μm^2^ s^−1^	Means *et al*. (2006)
Cytosolic buffer – parvalbumin			
Diffusion coefficient	*D*_B,cyt_	90 μm^2^ s^−1^	Bortolozzi *et al*. (2008)
Diffusion coefficient	*D*_CaB, cyt_	90 μm^2^ s^−1^	As for buffer
Forward binding constant	*K*_on_	18.5 μm ^−1^ s^−1^	Bortolozzi *et al*. (2008)
Backward binding constant	*K*_off_	0.95 s^−1^	Bortolozzi *et al*. (2008)
IP_3_R			
ER Ca^2+^concentration	*C*_Ca,er_	0.25 mm	Means *et al*. (2006)
Maximum channel current	*J*_0_	3.3 × 10^−19^ mol s^−1^	Tu *et al*. (2005); Means *et al*. (2006)

Rate constants and diffusion coefficients were obtained from Means *et al*. (2006) and Bortolozzi *et al*. (2008) and reported as measured in the cytoplasm of cells.

#### Computation

Equation (1) was solved for a regular voxel grid in Cartesian space (Table 1) using a first order finite difference solver. A sufficiently large volume was used such that edge effects are negligible, with the condition being that *C*_Ca,cyt_ did not exceed a level 1% above baseline at the edges (excluding the edge containing the IP_3_Rs), except where we intentionally simulated compressed cells. The Courant–Freidcrichs–Lewy condition:

(5) $$\frac{2D_{s}dt}{dx^{2}}<1$$

was obeyed for each species, where *dt* is the simulation time step and *dx* is the linear size of a voxel. Simulation was conducted using bespoke C code employing Intel AVX‐2 vector extensions. The Python language was used to process and analyse simulation results.

#### Boundary conditions

Our model was initialized with homogeneous Ca^2+^ and buffer concentrations as given in Table 1, with *C*_CaB,cyt_ being initialized to equilibrium values. A zero flux boundary condition was applied to the edges of the cuboidal simulation volume.

#### Simulation verification

Our simulation code was compared with analytical expressions for simplified test cases of 3D diffusion from a point source and for the microdomain profile of an isolated Ca^2+^ source in a strongly buffered environment.

#### Analytical diffusion

Equation (6) gives the analytical form of the concentration profile for a slug of mass *M* released at position *r* = 0 and time *t* = 0, where *r* is scalar radius from the origin (Balluffi *et al*. 2005).

(6) $$C\left(r,\;t\right)=\frac{M}{{\left(4\;D\pi t\right)}^{3/2}}\exp\left(-\frac{r^{2}}{4Dt}\right)$$

**Figure 3:**
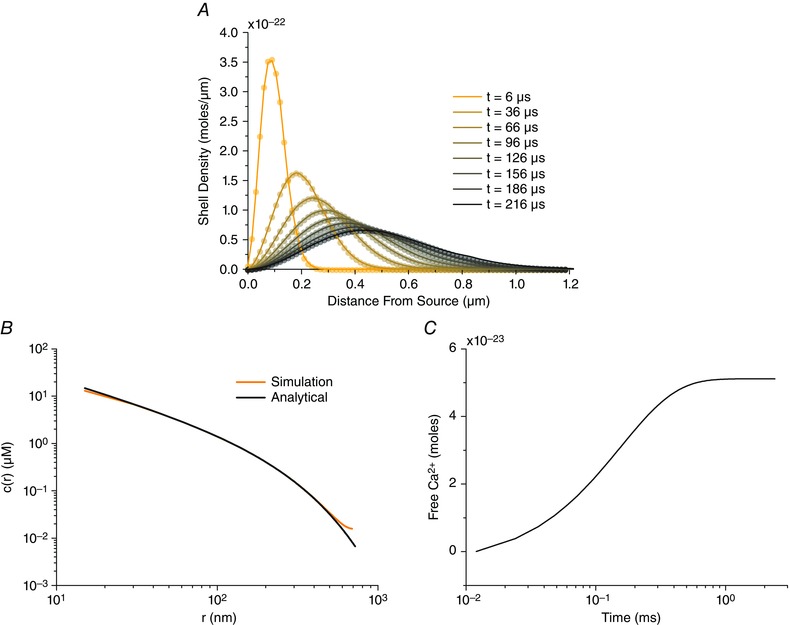
**IP_3_‐evoked Ca^2+^ release simulation** *A*, shell density plotted against distance from source (i.e. IP_3_R). Theoretical (analytical test case; lines) plots of shell density for a slug of mass released at the origin shows good agreement with our simulated results (circles). *B*, Ca^2+^ concentration as a function of radial distance from IP_3_Rs. When equilibrated, the Ca^2+^ profile may be approximated by an analytical case (black line), which compares accurately to an examination of our simulation (yellow line). *C*, free Ca^2+^ concentration at an IP_3_R as a function of time from receptor opening. The total cytosolic Ca^2+^ rises immediately after IP_3_R opening then plateaus as the current flowing through IP_3_R and cytosolic Ca^2+^ buffering reaches equilibrium (∼0.1 ms). See Methods and Table 1 for equations, source references and parameters used to generate the figures.

The Ca^2+^ diffusion component of our simulation was initialized with a volume of 1.68 μm on a side, with Ca^2+^ parameters as per Table 1, and with zero Ca^2+^ concentration except in the central voxel, which was set to 1 m. Figure 3 *A* and *B* illustrates the good agreement between our simulation and the analytical case.

#### Analytical microdomain profile

An analytical solution for the equilibrium concentration profile, at distance *r* from an isolated source of constant current *J_Ca_* in an isotropic, exists for an inexhaustible buffer that only forward binds (known as the excess buffer approximation; Smith, 1996):

(7) c(r)=c∞+J Ca 2πD Ca e−r/λr

where *c*_∞_ is the Ca^2+^ concentration far from the source – that is the equilibrium level, γ = (*Dτ*)^0.5^ is a space constant and τ = 1/(*K*_on_ *B*) is the mean capture time of the buffer, of concentration *B*. Our simulation was configured to match, as well as possible, this analytical case. Specifically parameters from Table 1 were used to simulate a single source, with the following modifications: *K*_Off_ was set to zero (forward binding only), ϕ_B,cyt_ from Eqn (2) was set to zero (inexhaustible buffer) and the concentration gradient was removed from Eqn (3) (constant current source). As a consequence of these changes any background Ca^2+^ was rapidly depleted in this model, so *c_∞_* was also set to zero to facilitate direct comparison. After running the model, the total quantity of free (unbuffered) cytosolic Ca^2+^ was examined (Fig. 3 *C*). This level rose rapidly, stabilizing to equilibrium in ∼0.1 ms. A comparison of Eqn (6) and the concentration profile of the model, once equilibrium is reached, is shown in Fig. 3. The responses were very similar, with the effect of quantized voxel sizes limiting the model concentration at small *r* and edge effects raising the concentration at high *r*.

#### Simulation parameters

Parameters for the simulation were chosen to be physiologically relevant, and are given in Table 1 along with references.

## Results

**Figure 4:**
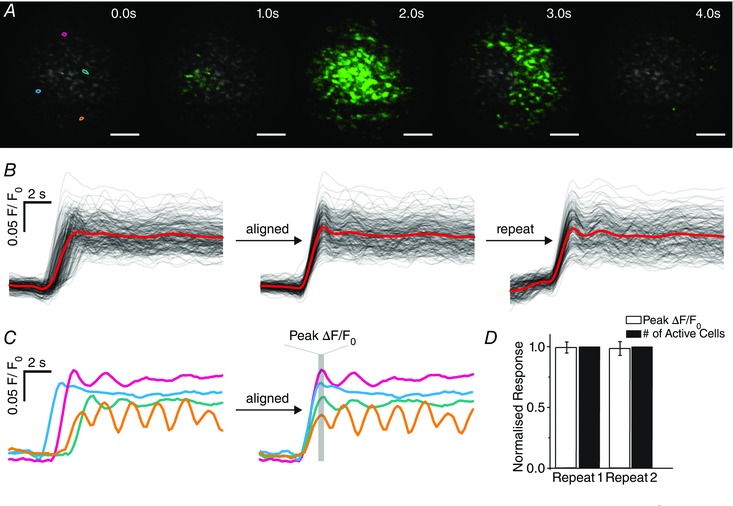
**Endothelial Ca^2+^ imaging in intact, pressurized (60 mmHg) arteries** *A*, time series Ca^2+^ images of endothelial cells showing the progression of the Ca^2+^ response evoked by ACh (100 μm; bath applied; 1 μm estimated at the vessel lumen) recorded at 60 mmHg. Images are composed of instantaneous Ca^2+^ activity (green) overlaid on standard deviation images (greyscale) indicative of total Ca^2+^ activity. The rise began in a distinct cluster of cells (see 1 s) and progressed from there. Each bright spot is an individual cell. Scale bar: 100 μm. *B*, ACh‐evoked Ca^2+^ signals from the same cells shown in *A*. In the left panel (unaligned data) the range of times for Ca^2+^ to increase for each of the ∼200 cells is approximately 4 s. The spread of responses resulted in mean data representing the data poorly. The position of the first peak of derivative signals was used to align Ca^2+^ signals. This procedure synchronizes the Ca^2+^ rises occurring in each due to the action of ACh (middle panel ‘aligned’) and illustrates total Ca^2+^ activity (thick red line) with increased clarity. On successive application of ACh (20 min reequilibration following wash), the response to ACh was reproducible (right panel). *C*, select examples of unaligned (left) and aligned (right) cellular Ca^2+^ responses from ROIs shown in *A*. The grey box illustrates the time point of measurements from aligned signals. *D*, summary data illustrating the reproducibility of Ca^2+^ responses upon repeat application (wash + 20 min reequilibration) with ACh. Data are presented as means ± SEM and normalized to control (*n* = 5; first ACh application, 1; not shown), *P* < 0.05 was considered significant.

**Figure 5:**
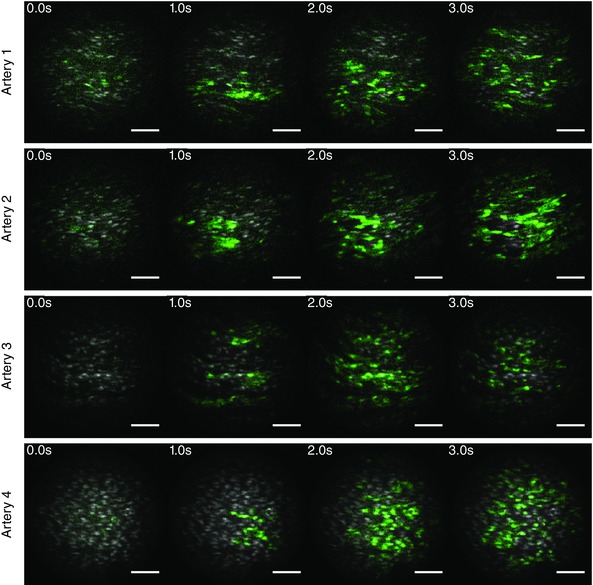
**Large‐scale, ACh‐evoked endothelial Ca^2+^ waves in four separate arteries** In each artery, a time series of Ca^2+^ images from the endothelium illustrates that large‐scale, ACh‐evoked (100 μm; bath application) Ca^2+^ waves originate in distinct clusters of cells and propagate from there across the endothelium. The images are composed of instantaneous Ca^2+^ activity (green) overlaid on standard deviation images (grayscale), which are indicative of total Ca^2+^ activity. Each bright spot is a single endothelial cell. Scale bars: 100 μm.

The GRIN microprobe inserted easily into the lumen of an artery (Fig. 1) delivered a field of view of 0.5 mm diameter and allowed a large number (∼200) of naturally connected endothelial cells to be imaged (Fig. 1 *I*) with subcellular resolution (∼4.5 μm; Fig. 2 *D*). In arteries pressurized to 60 mmHg, activation of the endothelium by extra‐luminal acetylcholine (ACh; 100 μm applied to the chamber, ∼1 μm estimated at the vessel lumen; 60 mmHg; online Supporting information, Movies S1 and S2) evoked reproducible rises in cytoplasmic Ca^2+^ concentration ([Ca^2+^]_c_) in the majority of cells in the field (Fig. 4 *A*). The response was composed of temporally distinct components. Initially, small spatial groupings of cells (macrodomain) activated (Fig. 4 *A*, Movie S2) in small regions of the endothelium and from which waves expanded (Fig. 4 *A*) to recruit the remaining majority of cells in the field of view (Figs 4 and 5).

As a result of the wave progression there was a large spread of times to first Ca^2+^ response (latency) in various cells (Fig. 4 *B* and *C*, left panels) which complicated summarizing and evaluating responses. Therefore, the temporal latency between individual Ca^2+^ traces (*F*/*F*_0_) was removed by aligning the signals to each other (using the initial peak in the first derivative of each signal) (Fig. 4 *B* and *C*, middle panels). This procedure removed heterogeneity resulting from the time to onset of individual signals and provided a clearer illustration of total Ca^2+^ activity. Upon consecutive stimulation with ACh, the response, as measured from the number of cells activated and the average peak change in *F*/*F*_0_, was approximately reproducible (Fig. 4 *D*, *n* = 5).

**Figure 6:**
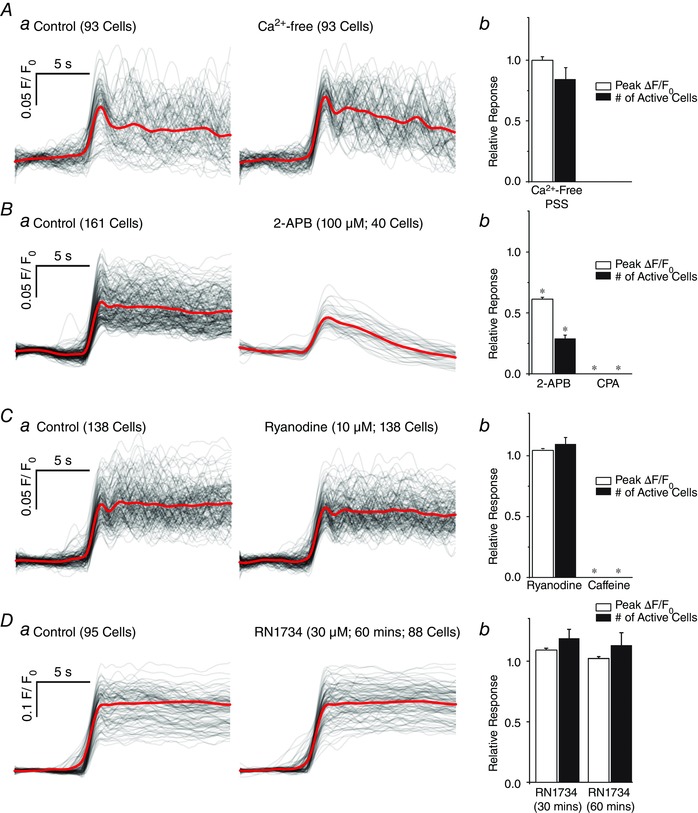
**The effects of Ca^2+^‐free bath solution and pharmacological activators and inhibitors on ACh‐evoked Ca^2+^ increases** Individual Ca^2+^ traces are shown on the left (*a*) and summarized data are presented as means ± SEM and normalized to control (no treatment, 1; not shown) on the right (*b*). *Aa* and *b*, the ACh‐evoked Ca^2+^ was comparable before and after the removal of Ca^2+^ from the bath solution (1 mm EGTA; *n* = 4). *Ba* and *b*, the ACh‐evoked Ca^2+^ rise was substantially reduced by aminoethoxydiphenyl borate (2‐APB, 100 μm, *n* = 3) and inhibited by the SERCA blocker cyclopiazonic acid (*Bb*; CPA, 10 μm, *n* = 3). *Ca* and *b*, ACh‐evoked responses persisted in the presence of ryanodine (10 μm; *n* = 3). Caffeine (10 mm; *n* = 3; *Cb*) failed to evoke any Ca^2+^ response. *Da* and *b*, ACh‐evoked responses were not reduced by the TRPV4 blocker RN1734 (30 μm; *n* = 3). Individual cellular Ca^2+^ traces are shown in grey with average overlaid in red. *P* < 0.05 was considered significant.

The Ca^2+^ rise evoked by ACh was approximately reproducible on successive application of ACh (Fig. 4 *D*; *n* = 5). The ACh‐evoked Ca^2+^ rise persisted in a Ca^2+^‐free bathing solution (*n* = 4), but was blocked by the SERCA inhibitor cyclopiazonic acid (10 μm; *n* = 3) and the IP_3_R blocker 2‐aminoethoxydiphenyl borate (100 μm; *n* = 3; Fig. 6). Caffeine (10 mm; *n* = 3) did not evoke a Ca^2+^ increase and the ACh‐evoked Ca^2+^ rise was unaltered by ryanodine (10 μm; *n* = 3). These results suggest that the Ca^2+^ rise originated from an IP_3_‐sensitive Ca^2+^ store and that RyRs play a minor role in Ca^2+^ signalling in the endothelium (Fig. 6). The TRPV4 antagonist RN1734 (30 μm; 30 min) did not decrease the ACh‐evoked endothelial response at 60 mmHg (Fig. 6 *D*) – as expected from previous findings in larger arteries (Hartmannsgruber *et al*. 2007; Loot *et al*. 2008).

**Figure 7:**
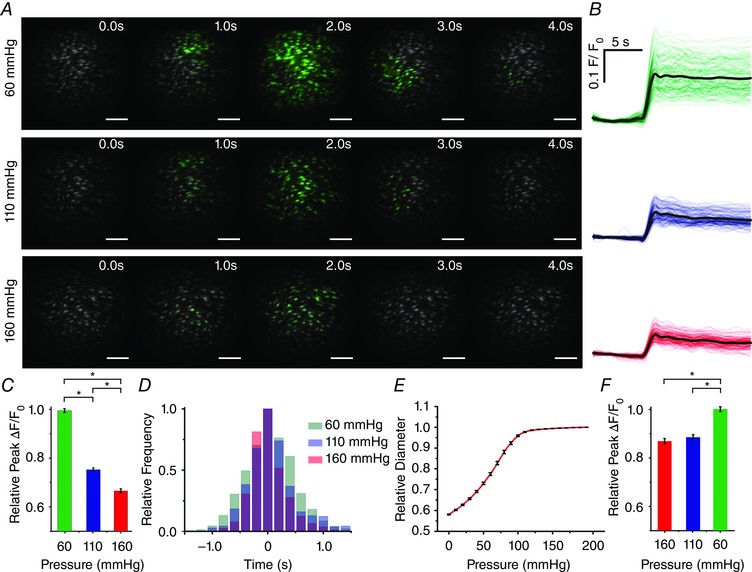
**Increased transmural pressure decreases endothelial Ca^2+^ signalling** *A*, ACh‐evoked Ca^2+^ images from endothelial cells at 60 mmHg (top), 110 mmHg (middle) and 160 mmHg (bottom). Ca^2+^ activity decreased as pressure increased in the three repeated activations of the same artery (20 min wash/re‐equilibration period between each ACh application). The data are shown as described in Fig. 2 *A*. Scale bar 100 μm. *B*, baseline corrected and aligned Ca^2+^ signals (average overlaid in black and shown in colour in the bottom panel) from the Ca^2+^ images shown in *A*. *C*, summary data illustrating the pressure‐dependent decrease in peak Δ*F*/*F*_0_ (*n* = 8, ±SEM). *D*, the temporal spread of cellular activation decreases with increased pressure. Note the fourth colour in the histogram (purple) arises from overlap of colours from the data in 110 mmHg (blue) and 160 mmHg (red). *E*, summary data illustrating a logistic change in vessel diameter as pressure is increased (from 0 to 200 mmHg; *n* = 3, ±SEM), measured using the Vessel Diameter plugin for ImageJ. Artery diameter was 1135 ± 110 μm at 60 mmHg, 1429 ± 92 μm at diameter at 110 mmHg and 1460 ± 83 μm at 160 mmHg (*n* = 3). *F*, summary data illustrating an increase in peak Δ*F*/*F*_0_ as pressure is decreased (*n* = 6, ±SEM).

Endothelial cells are stimulated almost constantly by extrinsic blood‐borne bioactive molecules amid a background of haemodynamic mechanical forces. The combined effect of chemical activators and mechanical force is presumably incorporated into the endothelium's biological response, but details are largely unknown. We addressed whether a change in endothelium‐dependent Ca^2+^ signalling, evoked by a bioactive molecule (ACh), occurs with variation in transmural pressure, and hence mechanical force. At 60 mmHg, ACh (100 μm) evoked asynchronous Ca^2+^ waves and oscillations that originated in macrodomains (Fig. 7 *A*; Movie S2). As transmural pressure increased (60 to 110 to 160 mmHg), both the amplitude of the Ca^2+^ rise that occurred in each cell and the temporal spread of total activation decreased (Fig. 7 *A–D*; Movie S3). The suppression of the amplitude of the Ca^2+^ signals reversed as pressure decreased (not shown). These results suggest that increased pressure in the intact artery suppressed IP_3_‐evoked Ca^2+^ signals. IP_3_R itself is not reported to be stretch sensitive, and mechanical activation of IP_3_‐generating processes is unlikely to explain our observations as transient increases in IP_3_ production and IP_3_‐evoked Ca^2+^ release occur typically (Jena *et al*. 1997).

**Figure 8:**
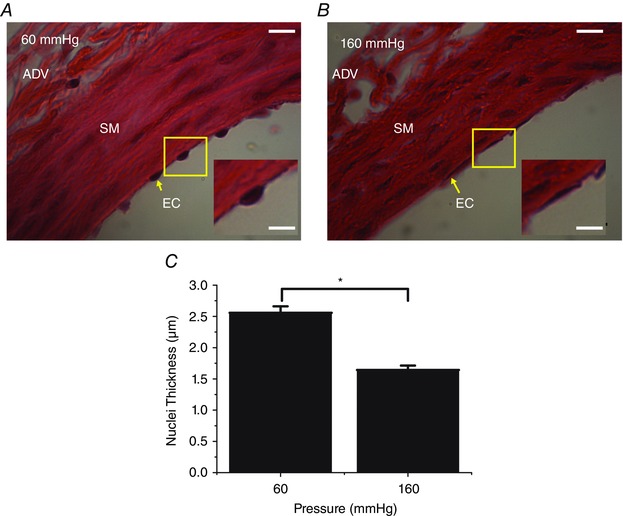
**Endothelial cell flattening as intraluminal pressure is increased** Arteries pressurized to 60 mmHg (*A*) and 160 mmHg (*B*) then fixed show flattening of the endothelial cells (EC) at higher (160 mmHg) pressure. The insets (*A* and *B*) show the yellow box expanded to illustrate the change in shape of single endothelial cells at each pressure. SM, smooth muscle; ADV, adventitia. Scale bars 10 μm main picture, inset 5 μm. *C*, average nuclei thickness (a measure of cell depth) at 60 mmHg and 160 mmHg. At 60 mmHg, *n* = 37 cells from 3 animals; 160 mmHg *n* = 35 cells from 3 animals (*P < 0.05).

The question arises as to how increased pressure suppressed IP_3_‐evoked Ca^2+^ signals. Significant changes in endothelial cell shape occurred with increased pressure (Fig. 8). Increased circumferential stretch, with increased pressure, elongated cells around the artery, and cell thickness was significantly decreased to maintain volume (Fig. 8 *A* and *B*). Increased transmural pressure may have also compressed the endothelial cells. The change in cell geometry is likely to alter Ca^2+^ dynamics in the endothelial cells at the microdomain level near IP_3_R. The effect of changes in cell geometry on IP_3_‐evoked Ca^2+^ signals was therefore considered further. To explore the effects of changes in the cells’ geometry on IP_3_‐mediated Ca^2+^ release at the relevant scale, which exists below functional optical microscopy, we computationally simulated events at an IP_3_R cluster.

IP_3_Rs are arranged in clusters and the sensitivity of IP_3_Rs to Ca^2+^ is such that the [Ca^2+^]_c_ in the microdomain of a single open IP_3_R (or cluster) may be high enough to activate nearby IP_3_Rs (or clusters)(Callamaras *et al*. 1998). This process of Ca^2+^ activation may become regenerative and carry a wave of activity across cells (Figs 4 and 5). In the transmission process, IP_3_R activity is exquisitely sensitive to distance between channels on the ER and to the cytosolic space between the ER and membrane structures, such as mitochondria and the plasma membrane (McGeown *et al*. 1996; Rizzuto *et al*. 1998; Mazel *et al*. 2009; Olson *et al*. 2010, 2012). These features (clustering, cytosolic space) create a local diffusive environment (i.e. microdomain) in which Ca^2+^ and Ca^2+^ buffers regulate the activity of IP_3_Rs and the release of Ca^2+^ from the ER due to the local [Ca^2+^]_c_.

**Figure 9:**
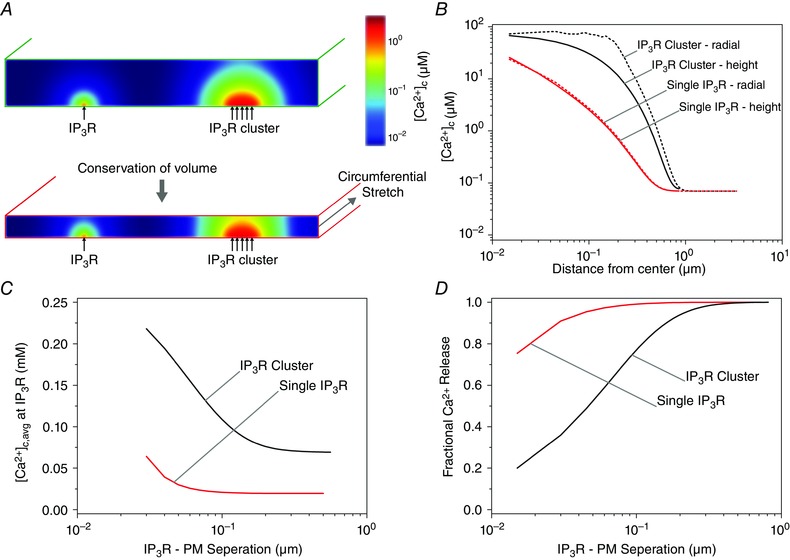
**Modelling endoplasmic reticulum Ca^2+^ release from solitary and clusters of IP_3_ receptors in cells subject to pressure‐induced stretch** *A*, cartoon illustrating the cytosolic Ca^2+^ distribution during Ca^2+^ release through a single IP_3_R and a cluster of IP_3_Rs as a cell (top, cell height: 0.57 μm) is stretched in the circumferential direction and thins to conserve volume (bottom, cell height: 0.27 μm). *B*, concentration profiles for [Ca^2+^]_c_ surrounding individual and clustered IP_3_Rs shows that the microdomain of elevated [Ca^2+^]_c_ is significantly larger for clusters of receptors. *C*, the average [Ca^2+^]_c_ in the vicinity of IP_3_Rs is sensitive to an increasing proximity between the ER and the plasma membrane (cell thinning). *D*, this results in a sensitivity of fractional Ca^2+^ release from internal stores that changes with receptor–plasma membrane distance (cell height) for single IP_3_R and a cluster of IP_3_Rs, with clusters having sensitivity over significantly larger cell heights, on the order of 500 nm.

The opening of a single IP_3_R introduced a point source of Ca^2+^ that equilibrated rapidly by diffusion and aggressive cytosolic buffering, to produce a localized, quasi‐static region of elevated [Ca^2+^]_c_ (microdomain; Fig. 9 *A* and *B*) around the channel. The Ca^2+^ concentration decreased sharply with distance from the IP_3_R with a characteristic scale (the distance at which [Ca^2+^] remains elevated by at least one order of magnitude) on the order of 100 nm (Fig. 9 *B*). The presence of many such point sources resulted in a greater [Ca^2+^]_c_ in the vicinity of IP_3_R clusters than isolated receptors (Fig. 9 *C*). The raised [Ca^2+^]_c_ that occurred as a result of IP_3_R clustering reduced the local ion gradient between the store ([Ca^2+^]_ER_) and the cytosol, and so reduced the entropic force driving Ca^2+^ release through the channel. In addition, the elevated [Ca^2+^]_c_ merged the microdomain of each IP_3_R into a unified, larger domain with scales approaching the 500 nm thickness typical of endothelial cells.

In wide, thin cells (e.g. endothelium) IP_3_Rs release Ca^2+^ into the cytosolic space between the ER and the cellular plasma membrane, where proximity of the IP_3_R to the cellular plasma membrane may create a restricted environment limiting the diffusion of Ca^2+^. The decreased cell height as a result of the change in cell shape (Fig. 8) reduces the distance between the IP_3_Rs and the opposing plasma membrane, restricting Ca^2+^ diffusion away from IP_3_R and, as a result, increasing the [Ca^2+^]_c_ at the receptors (Fig. 9 *A* and *C*). Thus, the decreased height reduced the total Ca^2+^ liberated from the ER by reducing the entropic force driving Ca^2+^ release (Fig. 9 *D*). In this way, a mechanical force (circumferential stretch) can reduce Ca^2+^ release via IP_3_R, in the absence of any force‐ or stretch‐sensitive molecular machinery. Notably, the clustering of IP_3_Rs extends this effect, when compared to isolated receptors, from sub‐100 nm thick cells to those with a 100–500 nm thickness typical of the endothelium (Fig. 9).

## Discussion

The endothelium's control over cardiovascular structure and function requires cooperation among endothelial cells to both sense and communicate stimuli efficiently with itself and other cell types (Ledoux *et al*. 2008; Behringer & Segal, 2012; Tran *et al*. 2012; Socha *et al*. 2012 *b*; Qian *et al*. 2013). The cooperative behaviour is not well understood because of the difficulties in studying large numbers of endothelial cells in a physiological configuration. The miniature, wide‐field GRIN imaging probe described permits endothelial Ca^2+^ activity from within an intact, pressurized artery to be observed. The microprobe bypasses the difficulties created by the artery wall in imaging blood vessels in a physiological configuration. Furthermore, the absence of biological tissue between the end of the probe and the endothelium minimized the light intensity required. The microprobe has a large depth‐of‐focus (141 μm) and delivers a 0.5 mm field of view to allow 200 cells to be viewed simultaneously.

The detection of chemical stimuli appears to be distributed among endothelial cells. In response to ACh, Ca^2+^ rises initiate in small regions of the endothelium and expand from these regions to recruit most of the cells in the field. The initiator regions (macrodomains) appear to be more sensitive to ACh. The path of the Ca^2+^ signal from the sensitive regions at times appeared poorly coordinated among endothelial cells – even chaotic. Nonetheless, the path and pattern were repeatable on successive application of ACh suggesting an encoded signal communicated among cells. The response to ACh was derived from an IP_3_‐mediated release of Ca^2+^ from the internal store. The Ca^2+^ rise persisted in a Ca^2+^‐free bath solution and was abolished by the SERCA pump inhibitor cyclopiazonic acid. The ACh‐evoked Ca^2+^ rise persisted in ryanodine and caffeine failed to evoke a Ca^2+^ rise. However, release was inhibited by the IP_3_R blocker 2‐APB. The transmission of the Ca^2+^ signal among endothelial cells may involve Ca^2+^ or IP_3_ or both.

In addition to receiving and communicating chemical stimuli, arteries are under permanent mechanical activation from shear stress and blood pressure. While cells often respond to the activation of mechanically sensitive components (e.g. via shear stress) with increased ion channel and cell activity, the mechanical response to pressure differs and endothelial activity is suppressed. For example, transient and chronic increases in pressure each inhibit endothelial nitric oxide‐mediated vasodilatation (Hishikawa *et al*. 1992; De Bruyn *et al*. 1994; Huang *et al*. 1998; Paniagua *et al*. 2000; Jurva *et al*. 2006; Phillips *et al*. 2011). How pressure may suppress endothelial activity is unresolved but increased reactive oxygen species may contribute (Huang *et al*. 1998; Vecchione *et al*. 2009; Zhao *et al*. 2015). Here we report that endothelial Ca^2+^ signalling is suppressed when pressure is elevated. The suppressed Ca^2+^ signalling will presumably result in reduced production of nitric oxide and reduced endothelium‐dependent dilatation (Hishikawa *et al*. 1992; Sun *et al*. 2001).

**Figure 10:**
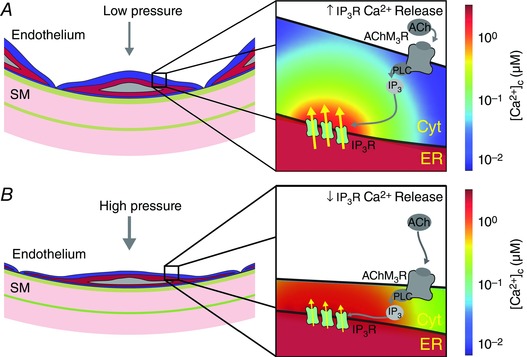
**Model of pressure‐dependent, geometric suppression of Ca^2+^ release through IP_3_Rs** As pressure is increased, from low (*A*) to high (*B*), arterial diameter increases and endothelial cells are stretched along the circumferential direction. In addition, the radial force of pressure acts upon the cells. To conserve volume, the height of endothelial cells decrease (*B*). The insets depict the processes leading to changes in ACh‐induced Ca^2+^ release in an endothelial cell at low (*A*, top) and high (*B*, bottom) pressure. At low pressure, activation of IP_3_R, after ACh application, results in Ca^2+^ release from the endoplasmic reticulum (ER). Ca^2+^ diffuses rapidly from the release site to maintain a gradient for continued release. The microdomains of Ca^2+^ operate over sizes (300 nm) comparable to the height of an endothelial cell (see text). When pressure is increased (*B*), the height of the cell decreases and so the distance between the ER and the plasma membrane (PM) also decreases. The decrease in ER–PM distance restricts the diffusion of Ca^2+^ from the release site (IP_3_Rs), effectively increasing the local Ca^2+^ microdomain concentration and decreasing the entropic force (indicated by size of yellow arrows) driving Ca^2+^ release through IP_3_Rs. The resultant effect is a decrease in Ca^2+^ release from the ER as pressure is increased. SM: smooth muscle.

The sensing system(s) responsible for detecting changes in pressure in the endothelium are poorly understood. Pressure‐evoked mechanical *deactivation* of ion channels has been proposed to account for some physiological responses (Hoffman *et al*. 2011; Bagher *et al*. 2012). We propose that changes in cell shape may be sufficient to underlie the detection of pressure‐dependent mechanical forces by the endothelium. The changes in geometric shape of the cell in response to pressure changes are substantial: endothelial cells may be flattened either by circumferential stretch, as the height of endothelial cells is reduced to maintain volume, or by compression due to the radial force of pressure or both. Such changes in the geometric shape of the cell will bring the endoplasmic reticulum and plasma membrane closer together and create a region of restricted diffusion (see also Hong *et al*. 2008; Qi *et al*. 2015). As shown in Fig. 9, the region of restricted diffusion changes the local diffusive environment surrounding IP_3_R clusters and results in a higher concentration of Ca^2+^ near the IP_3_R microdomain. This higher concentration results in a reduced Ca^2+^ gradient between the store and the cytosol. As a result, the entropic force driving Ca^2+^ release is reduced and so the amplitude of IP_3_‐evoked Ca^2+^ signals is also reduced (Fig. 10). Reduced IP_3_‐evoked Ca^2+^ release may explain the suppression of artery level endothelium‐derived responses at high transmural pressure (De Bruyn *et al*. 1994; Huang *et al*. 1998; Paniagua *et al*. 2000; Zhao *et al*. 2015). A specific force‐ or stretch‐sensitive component may not be required, but rather changes in cell shape may alter the local concentrations of second messengers (e.g. Ca^2+^) within the cell to modulate the overall response of the cell.

The pressure‐sensitive change in IP_3_‐evoked Ca^2+^ signalling provides an additional level of complexity and subtly to mechanosensitive signalling, which may induce further signals to generate a functional response. For example, the changes in [Ca^2+^]_c_ as a result of geometric modulation of IP_3_‐evoked Ca^2+^ release may modulate the activity of plasma membrane‐located Ca^2+^‐sensitive ion channels to explain, at least in part, some contradictory observations of apparent mechanical activation (shear stress, Strotmann *et al*. 2000; Alessandri‐Haber *et al*. 2003; Loukin *et al*. 2010) and inhibition (pressure increases, Bagher *et al*. 2012) of ion channels. In the latter study, when pressure decreased from 80 mmHg to 5 mmHg, the frequency of transient localized endothelial Ca^2+^ rises increased (Bagher *et al*. 2012). The authors attributed the Ca^2+^ changes to influx via TRPV4. The mechanosensor responsible for the pressure‐induced change in TRPV4 activity was unclear but the authors suggested reduced radial compression of endothelial cells may activate the channel (Bagher *et al*. 2012). TRPV4 is Ca^2+^ sensitive and activated by increases in [Ca^2+^]_c_ (Strotmann *et al*. 2003). Interestingly, in their study Bagher *et al*. (2012) found the local endothelial Ca^2+^ events studied were blocked fully by a phospholipase C inhibitor (U‐73122), and an IP_3_R blocker (xestospongin C) almost abolished Ca^2+^ events at 80 mmHg and significantly inhibited responses at 5 mmHg. These results suggest that IP_3_‐mediated Ca^2+^ release may contribute significantly to the spontaneous Ca^2+^ events. Geometric regulation of IP_3_‐evoked Ca^2+^ release may provide the pressure sensor for the change in TRPV4 activity.

Endothelial cells are acknowledged to be sensitive to their geometry and changes are known to regulate proliferation and cell death (Chen *et al*. 1997). Changes in cell shape alter tension in the cell membrane to modify components within membranes (Sheetz, 1993; Mederos y Schnitzler *et al*. 2008) with consequences for cell behaviour (Mossman *et al*. 2005). Alternatively, changes in links between the plasma membrane and cytoskeleton or mechanosensitive ion channels may detect changes in membrane tension (Patel *et al*. 2001). Several proteins also have domains (BAR or ALPS) found in membranes that are curved and may contribute to detecting cell shape changes. The extent of membrane binding of these protein domains changes with membrane curvature (Zhao *et al*. 2011). The protein domains may either induce or are sensitive to membrane curvature (Zimmerberg & Kozlov, 2006). Guanine nucleotide exchange factors and GTPase activating proteins with curvature sensing protein domains may alter the activity of GTPases in a tension‐dependent manner (de Kreuk *et al*. 2011) to explain geometry sensitivity of cells. We propose changes in cell geometry may also alter ion diffusional gradients, which act then to sense stimuli and evoke vascular responses.

For normal function, cells in the vascular wall must act in a coordinated manner. The spatial relationships among extracellular chemical stimuli, cellular architecture and Ca^2+^ signals are central to signal processing and the coordinated behaviour. Indeed, although ‘sensing’ may occur on the microdomain scale, the macroscopic configuration of the intact endothelium is essential for physiological mechanotransduction. The detection of mechanical forces like pressure is critically dependent on the organization of the sensing proteins and the contact of cells with other cells. In other investigations on mechanotransduction, a single type of protein was found to evoke different responses when either organized in 3D filaments or arranged on a flat surface (Cukierman *et al*. 2001). Some types of mechanosensitivity seen in isolated single cells were lost when cells are in contact with one another (Yeung *et al*. 2005). Our results in intact arteries in a physiological configuration suggest that mechanical activation by pressure may be merged into the agonist‐evoked response in the endothelium. The pressure dependence of Ca^2+^ release provides a mechanism for transmural pressure to be encoded and integrated to a common, coordinated output without imposing a requirement for mechanosensitivity on any of the molecular machinery in the signalling pathway. The time course of this response would be rapid and may form an early event in an altered mechanical environment. Furthermore, our results suggest how hypertensive conditions may limit the ability of cells to engage in Ca^2+^ signalling, offering insight into the associated degeneration of endothelial response (Luscher & Vanhoutte, 1986; Wallace *et al*. 2007) and the role that IP_3_R clustering may play.

## Additional information

### Competing interests

None.

### Author contributions

All authors have approved the final version of the manuscript and agree to be accountable for all aspects of the work. All persons designated as authors qualify for authorship, and all those who qualify for authorship are listed. The manuscript was prepared and written with contributions from all authors. All authors approved the final version of the manuscript.

### Funding

This work was funded by the Wellcome Trust (092292/Z/10/Z) and British Heart Foundation (PG/11/70/29086 and Research Excellence Award (Edinburgh/Durham University)) and EPSRC; their support is gratefully acknowledged.

## Supporting information


**Movie S1.** ACh‐evoked endothelial Ca^2+^ signals in a pressurised (60 mmHg) rat carotid artery. Activation of IP_3_‐evoked endothelial Ca^2+^ signals, by bath application of ACh (100 μM), causes a rise in cytoplasmic Ca^2+^ concentration in the majority of cells across the field of view. The movie is composed of raw data, to which a 5‐frame rolling average has been applied, that has been linearly contrast adjusted for visualisation. Data was acquired at 5 Hz and the scale bar is 100 μm.

**Movie S2**. ACh activated IP_3_‐evoked endothelial Ca^2+^ signals which were recorded from inside pressurized arteries. At 60 mmHg, ACh caused an initial burst of Ca^2+^ increases that propagate as waves. As ACh‐evoked Ca^2+^ waves expanded collision between adjacent cells occurred and wave annihilation occurred. As a result of multiple waves progressions and annihilation events, complex spatiotemporal patterns of Ca^2+^ signaling developed. The movie is composed of a sequential subtraction of the temporally smoothed Ca^2+^ activity (green) overlaid on standard deviation image (grayscale). Data was acquired at 5 Hz and the scale bar is 100 μm.

**Movie S3**. ACh activated IP_3_‐evoked Ca^2+^ signals were significantly attenuated as the artery transmural pressure increased from 60 mmHg to 110 mmHg to 160 mmHg. After each application of ACh the bath solution was washed with >20 times the bath volume, and the artery was allowed to re‐equilibrate for 20 minutes before the next pressure change and ACh addition. The movie corresponds to Ca^2+^ traces and time‐series data shown in Figure 7A and Figure 7B respectively. The movie is a time series of Ca^2+^ wave activity (green) overlaid on standard deviation images (STDev) (grayscale). Note that STDev images only show cells that exhibit Ca^2+^ activity. Data was acquired at 5 Hz and the scale bar corresponds to 100 μm.

## References

[tjp6918-bib-0001] Alessandri‐Haber N , Yeh JJ , Boyd AE , Parada CA , Chen X , Reichling DB & Levine JD (2003). Hypotonicity induces TRPV4‐mediated nociception in rat. Neuron 39, 497–511. 12895423 10.1016/s0896-6273(03)00462-8

[tjp6918-bib-0002] Angus JA , Campbell GR , Cocks TM & Manderson JA (1983). Vasodilatation by acetylcholine is endothelium‐dependent: a study by sonomicrometry in canine femoral artery in vivo. J Physiol 344, 209–222. 6197520 10.1113/jphysiol.1983.sp014934 PMC1193835

[tjp6918-bib-0003] Bagher P , Beleznai T , Kansui Y , Mitchell R , Garland CJ & Dora KA (2012). Low intravascular pressure activates endothelial cell TRPV4 channels, local Ca^2+^ events, and IKCa channels, reducing arteriolar tone. Proc Natl Acad Sci USA 109, 18174–18179. 23071308 10.1073/pnas.1211946109 PMC3497745

[tjp6918-bib-0004] Balluffi R , Allen S & Carter W (2005). Kinetics of Materials. John Wiley and Sons, Hoboken.

[tjp6918-bib-0005] Behringer EJ & Segal SS (2012). Tuning electrical conduction along endothelial tubes of resistance arteries through Ca^2+^‐activated K^+^ channels. Circ Res 110, 1311–1321. 22492531 10.1161/CIRCRESAHA.111.262592 PMC3467972

[tjp6918-bib-0006] Behringer EJ & Segal SS (2015). Membrane potential governs calcium influx into microvascular endothelium: Integral role for muscarinic receptor activation. J Physiol 593, 4531–4548. 26260126 10.1113/JP271102 PMC4606535

[tjp6918-bib-0007] Billaud M , Lohman AW , Johnstone SR , Biwer LA , Mutchler S & Isakson BE (2014). Regulation of cellular communication by signaling microdomains in the blood vessel wall. Pharmacol Rev 66, 513–569. 24671377 10.1124/pr.112.007351 PMC3973613

[tjp6918-bib-0008] Bortolozzi M , Lelli A & Mammano F (2008). Calcium microdomains at presynaptic active zones of vertebrate hair cells unmasked by stochastic deconvolution. Cell Calcium 44, 158–168. 18249440 10.1016/j.ceca.2007.11.007

[tjp6918-bib-0009] Bradley KN , Currie S , MacMillan D , Muir TC & McCarron JG (2003). Cyclic ADP‐ribose increases Ca^2+^ removal in smooth muscle. J Cell Sci 116, 4291–4306. 12966165 10.1242/jcs.00713

[tjp6918-bib-0010] Bubolz AH , Mendoza SA , Zheng X , Zinkevich NS , Li R , Gutterman DD & Zhang DX (2012). Activation of endothelial TRPV4 channels mediates flow‐induced dilation in human coronary arterioles: role of Ca^2+^ entry and mitochondrial ROS signaling. Am J Physiol Heart Circ Physiol 302, H634–642. 22140047 10.1152/ajpheart.00717.2011 PMC3353785

[tjp6918-bib-0011] Callamaras N , Marchant JS , Sun XP & Parker I (1998). Activation and co‐ordination of InsP_3_‐mediated elementary Ca^2+^ events during global Ca^2+^ signals in *Xenopus* oocytes. J Physiol 509, 81–91. 9547383 10.1111/j.1469-7793.1998.081bo.x PMC2230929

[tjp6918-bib-0012] Chataigneau T , Feletou M , Duhault J & Vanhoutte PM (1998 *a*). Epoxyeicosatrienoic acids, potassium channel blockers and endothelium‐dependent hyperpolarization in the guinea‐pig carotid artery. Br J Pharmacol 123, 574–580. 9504399 10.1038/sj.bjp.0701629 PMC1565190

[tjp6918-bib-0013] Chataigneau T , Feletou M , Thollon C , Villeneuve N , Vilaine JP , Duhault J & Vanhoutte PM (1998 *b*). Cannabinoid CB1 receptor and endothelium‐dependent hyperpolarization in guinea‐pig carotid, rat mesenteric and porcine coronary arteries. Br J Pharmacol 123, 968–974. 9535027 10.1038/sj.bjp.0701690 PMC1565243

[tjp6918-bib-0014] Chen CS , Mrksich M , Huang S , Whitesides GM & Ingber DE (1997). Geometric control of cell life and death. Science 276, 1425–1428. 9162012 10.1126/science.276.5317.1425

[tjp6918-bib-0015] Chien S (2007). Mechanotransduction and endothelial cell homeostasis: the wisdom of the cell. Am J Physiol Heart Circ Physiol 292, H1209–1224. 17098825 10.1152/ajpheart.01047.2006

[tjp6918-bib-0016] Corey DP , Garcia‐Anoveros J , Holt JR , Kwan KY , Lin SY , Vollrath MA , Amalfitano A , Cheung EL , Derfler BH , Duggan A , Geleoc GS , Gray PA , Hoffman MP , Rehm HL , Tamasauskas D & Zhang DS (2004). TRPA1 is a candidate for the mechanosensitive transduction channel of vertebrate hair cells. Nature 432, 723–730. 15483558 10.1038/nature03066

[tjp6918-bib-0017] Craig J & Martin W (2012). Dominance of flow‐mediated constriction over flow‐mediated dilatation in the rat carotid artery. Br J Pharmacol 167, 527–536. 22537086 10.1111/j.1476-5381.2012.02006.x PMC3449258

[tjp6918-bib-0018] Cukierman E , Pankov R , Stevens DR & Yamada KM (2001). Taking cell‐matrix adhesions to the third dimension. Science 294, 1708–1712. 11721053 10.1126/science.1064829

[tjp6918-bib-0019] De Bruyn VH , Nuno DW , Cappelli‐Bigazzi M , Dole WP & Lamping KG (1994). Effect of acute hypertension in the coronary circulation: role of mechanical factors and oxygen radicals. J Hypertens 12, 163–172. 8021468

[tjp6918-bib-0020] de Kreuk BJ , Nethe M , Fernandez‐Borja M , Anthony EC , Hensbergen PJ , Deelder AM , Plomann M & Hordijk PL (2011). The F‐BAR domain protein PACSIN2 associates with Rac1 and regulates cell spreading and migration. J Cell Sci 124, 2375–2388. 21693584 10.1242/jcs.080630

[tjp6918-bib-0021] Deanfield JE , Halcox JP & Rabelink TJ (2007). Endothelial function and dysfunction: testing and clinical relevance. Circulation 115, 1285–1295. 17353456 10.1161/CIRCULATIONAHA.106.652859

[tjp6918-bib-0022] Dedman A , Sharif‐Naeini R , Folgering JH , Duprat F , Patel A & Honore E (2009). The mechano‐gated K_2P_ channel TREK‐1. Eur Biophys J 38, 293–303. 18369610 10.1007/s00249-008-0318-8

[tjp6918-bib-0023] Dunn WR , Wellman GC & Bevan JA (1994). Enhanced resistance artery sensitivity to agonists under isobaric compared with isometric conditions. Am J Physiol Heart Circ Physiol 266, H147–155. 10.1152/ajpheart.1994.266.1.H147 8304495

[tjp6918-bib-0024] Duza T & Sarelius IH (2004). Localized transient increases in endothelial cell Ca^2+^ in arterioles in situ: implications for coordination of vascular function. Am J Physiol Heart Circ Physiol 286, H2322–2331. 14962843 10.1152/ajpheart.00006.2004

[tjp6918-bib-0025] Earley S & Brayden JE (2015). Transient receptor potential channels in the vasculature. Physiol Rev 95, 645–690. 25834234 10.1152/physrev.00026.2014 PMC4551213

[tjp6918-bib-0026] Edelstein A , Amodaj N , Hoover K , Vale R & Stuurman N (2010). Computer control of microscopes using microManager. In *Current Protocols in Molecular Biology*, ed. Ausubel MF *et al*., Ch. 14, Unit 14.20. 10.1002/0471142727.mb1420s92 PMC3065365 20890901

[tjp6918-bib-0027] Falcone JC , Kuo L & Meininger GA (1993). Endothelial cell calcium increases during flow‐induced dilation in isolated arterioles. Am J Physiol Heart Circ Physiol 264, H653–659. 10.1152/ajpheart.1993.264.2.H653 8447477

[tjp6918-bib-0028] Faraci FM & Heistad DD (1990). Regulation of large cerebral arteries and cerebral microvascular pressure. Circ Res 66, 8–17. 2403863 10.1161/01.res.66.1.8

[tjp6918-bib-0029] Faraci FM , Orgren K & Heistad DD (1994). Impaired relaxation of the carotid artery during activation of ATP‐sensitive potassium channels in atherosclerotic monkeys. Stroke 25, 178–182. 8266368 10.1161/01.str.25.1.178

[tjp6918-bib-0030] Fischer MJ , Uchida S & Messlinger K (2010). Measurement of meningeal blood vessel diameter in vivo with a plug‐in for ImageJ. Microvasc Res 80, 258–266. 20406650 10.1016/j.mvr.2010.04.004

[tjp6918-bib-0031] Flusberg BA , Nimmerjahn A , Cocker ED , Mukamel EA , Barretto RP , Ko TH , Burns LD , Jung JC & Schnitzer MJ (2008). High‐speed, miniaturized fluorescence microscopy in freely moving mice. Nat Methods 5, 935–938. 18836457 10.1038/nmeth.1256 PMC2828344

[tjp6918-bib-0032] Francis M , Qian X , Charbel C , Ledoux J , Parker JC & Taylor MS (2012). Automated region of interest analysis of dynamic Ca^2+^ signals in image sequences. Am J Physiol Cell Physiol 303, C236–243. 22538238 10.1152/ajpcell.00016.2012 PMC3423022

[tjp6918-bib-0033] Gunduz F , Meiselman HJ & Baskurt OK (2008). High intravascular pressure attenuates vascular dilation responses of small mesenteric arteries in the rat. Circ J 72, 482–486. 18296850 10.1253/circj.72.482

[tjp6918-bib-0034] Hartmannsgruber V , Heyken WT , Kacik M , Kaistha A , Grgic I , Harteneck C , Liedtke W , Hoyer J & Kohler R (2007). Arterial response to shear stress critically depends on endothelial TRPV4 expression. PLoS One 2, e827. 17786199 10.1371/journal.pone.0000827 PMC1959246

[tjp6918-bib-0035] Hishikawa K , Nakaki T , Suzuki H , Saruta T & Kato R (1992). Transmural pressure inhibits nitric oxide release from human endothelial cells. Eur J Pharmacol 215, 329–331. 1396999 10.1016/0014-2999(92)90051-5

[tjp6918-bib-0036] Hoffman BD , Grashoff C & Schwartz MA (2011). Dynamic molecular processes mediate cellular mechanotransduction. Nature 475, 316–323. 21776077 10.1038/nature10316 PMC6449687

[tjp6918-bib-0037] Hong D , Jaron D , Buerk DG & Barbee KA (2008). Transport‐dependent calcium signaling in spatially segregated cellular caveolar domains. Am J Physiol Cell Physiol 294, C856–866. 18160488 10.1152/ajpcell.00278.2007

[tjp6918-bib-0038] Huang A , Sun D , Kaley G & Koller A (1998). Superoxide released to high intra‐arteriolar pressure reduces nitric oxide‐mediated shear stress‐ and agonist‐induced dilations. Circ Res 83, 960–965. 9797346 10.1161/01.res.83.9.960

[tjp6918-bib-0039] Huang TY , Chu TF , Chen HI & Jen CJ (2000). Heterogeneity of [Ca^2+^]_i_ signaling in intact rat aortic endothelium. FASEB J 14, 797–804. 10744636 10.1096/fasebj.14.5.797

[tjp6918-bib-0040] Janssen DA , Hoenderop JG , Jansen KC , Kemp AW , Heesakkers JP & Schalken JA (2011). The mechanoreceptor TRPV4 is localized in adherence junctions of the human bladder urothelium: a morphological study. J Urol 186, 1121–1127. 21784462 10.1016/j.juro.2011.04.107

[tjp6918-bib-0041] Jena M , Minore JF & O'Neill WC (1997). A volume‐sensitive, IP_3_‐insensitive Ca^2+^ store in vascular endothelial cells. Am J Physiol Cell Physiol 273, C316–322. 10.1152/ajpcell.1997.273.1.C316 9252470

[tjp6918-bib-0042] Jurva JW , Phillips SA , Syed AQ , Syed AY , Pitt S , Weaver A & Gutterman DD (2006). The effect of exertional hypertension evoked by weight lifting on vascular endothelial function. J Am Coll Cardiol 48, 588–589. 16875990 10.1016/j.jacc.2006.05.004

[tjp6918-bib-0043] Kim P , Chung E , Yamashita H , Hung KE , Mizoguchi A , Kucherlapati R , Fukumura D , Jain RK & Yun SH (2010). In vivo wide‐area cellular imaging by side‐view endomicroscopy. Nat Methods 7, 303–305. 20228814 10.1038/nmeth.1440 PMC2849759

[tjp6918-bib-0044] Knudsen HL & Frangos JA (1997). Role of cytoskeleton in shear stress‐induced endothelial nitric oxide production. Am J Physiol Heart Circ Physiol 273, H347–355. 10.1152/ajpheart.1997.273.1.H347 9249510

[tjp6918-bib-0045] Kohler R , Heyken WT , Heinau P , Schubert R , Si H , Kacik M , Busch C , Grgic I , Maier T & Hoyer J (2006). Evidence for a functional role of endothelial transient receptor potential V4 in shear stress‐induced vasodilatation. Arterioscler Thromb Vasc Biol 26, 1495–1502. 16675722 10.1161/01.ATV.0000225698.36212.6a

[tjp6918-bib-0046] Kusche‐Vihrog K , Jeggle P & Oberleithner H (2014). The role of ENaC in vascular endothelium. Pflugers Arch 466, 851–859. 24046153 10.1007/s00424-013-1356-3

[tjp6918-bib-0047] Lamping KG & Faraci FM (2001). Role of sex differences and effects of endothelial NO synthase deficiency in responses of carotid arteries to serotonin. Arterioscler Thromb Vasc Biol 21, 523–528. 11304467 10.1161/01.atv.21.4.523

[tjp6918-bib-0048] Ledoux J , Taylor MS , Bonev AD , Hannah RM , Solodushko V , Shui B , Tallini Y , Kotlikoff MI & Nelson MT (2008). Functional architecture of inositol 1,4,5‐trisphosphate signaling in restricted spaces of myoendothelial projections. Proc Natl Acad Sci USA 105, 9627–9632. 18621682 10.1073/pnas.0801963105 PMC2474537

[tjp6918-bib-0049] Li J , Hou B , Tumova S , Muraki K , Bruns A , Ludlow MJ , Sedo A , Hyman AJ , McKeown L , Young RS , Yuldasheva NY , Majeed Y , Wilson LA , Rode B , Bailey MA , Kim HR , Fu Z , Carter DA , Bilton J , Imrie H , Ajuh P , Dear TN , Cubbon RM , Kearney MT , Prasad RK , Evans PC , Ainscough JF & Beech DJ (2014). Piezo1 integration of vascular architecture with physiological force. Nature 515, 279–282. 25119035 10.1038/nature13701 PMC4230887

[tjp6918-bib-0050] Loot AE , Popp R , Fisslthaler B , Vriens J , Nilius B & Fleming I (2008). Role of cytochrome P450‐dependent transient receptor potential V4 activation in flow‐induced vasodilatation. Cardiovasc Res 80, 445–452. 18682435 10.1093/cvr/cvn207

[tjp6918-bib-0051] Loukin S , Zhou X , Su Z , Saimi Y & Kung C (2010). Wild‐type and brachyolmia‐causing mutant TRPV4 channels respond directly to stretch force. J Biol Chem 285, 27176–27181. 20605796 10.1074/jbc.M110.143370 PMC2930716

[tjp6918-bib-0052] Luscher TF & Vanhoutte PM (1986). Endothelium‐dependent contractions to acetylcholine in the aorta of the spontaneously hypertensive rat. Hypertension 8, 344–348. 2870025 10.1161/01.hyp.8.4.344

[tjp6918-bib-0053] McCarron JG , Chalmers S , MacMillan D & Olson ML (2010). Agonist‐evoked Ca^2+^ wave progression requires Ca^2+^ and IP_3_ . J Cell Physiol 224, 334–344. 20432430 10.1002/jcp.22103 PMC3947531

[tjp6918-bib-0054] McGeown JG , Drummond RM , McCarron JG & Fay FS (1996). The temporal profile of calcium transients in voltage clamped gastric myocytes from *Bufo marinus* . J Physiol 497, 321–336. 8961178 10.1113/jphysiol.1996.sp021771 PMC1160987

[tjp6918-bib-0055] Marchenko SM & Sage SO (2000). Effects of shear stress on [Ca^2+^]_i_ and membrane potential of vascular endothelium of intact rat blood vessels. Exp Physiol 85, 43–48. 10662891

[tjp6918-bib-0056] Maroto R , Raso A , Wood TG , Kurosky A , Martinac B & Hamill OP (2005). TRPC1 forms the stretch‐activated cation channel in vertebrate cells. Nat Cell Biol 7, 179–185. 15665854 10.1038/ncb1218

[tjp6918-bib-0057] Mazel T , Raymond R , Raymond‐Stintz M , Jett S & Wilson BS (2009). Stochastic modeling of calcium in 3D geometry. Biophys J 96, 1691–1706. 19254531 10.1016/j.bpj.2008.10.066 PMC2996128

[tjp6918-bib-0058] Mchedlishvili G (1986). Arterial Behavior and Blood Circulation in the Brain. Plenum Publishing Corp, New York.

[tjp6918-bib-0059] Means S , Smith AJ , Shepherd J , Shadid J , Fowler J , Wojcikiewicz RJ , Mazel T , Smith GD & Wilson BS (2006). Reaction diffusion modeling of calcium dynamics with realistic ER geometry. Biophys J 91, 537–557. 16617072 10.1529/biophysj.105.075036 PMC1483115

[tjp6918-bib-0060] Mederos Y , Schnitzler M , Storch U , Meibers S , Nurwakagari P , Breit A , Essin K , Gollasch M & Gudermann T (2008). Gq‐coupled receptors as mechanosensors mediating myogenic vasoconstriction. EMBO J 27, 3092–3103. 18987636 10.1038/emboj.2008.233 PMC2599876

[tjp6918-bib-0061] Mendoza SA , Fang J , Gutterman DD , Wilcox DA , Bubolz AH , Li R , Suzuki M & Zhang DX (2010). TRPV4‐mediated endothelial Ca^2+^ influx and vasodilation in response to shear stress. Am J Physiol Heart Circ Physiol 298, H466–476. 19966050 10.1152/ajpheart.00854.2009 PMC2822567

[tjp6918-bib-0062] Moccia F , Berra‐Romani R & Tanzi F (2012). Update on vascular endothelial Ca^2+^ signalling: A tale of ion channels, pumps and transporters. World J Biol Chem 3, 127–158. 22905291 10.4331/wjbc.v3.i7.127 PMC3421132

[tjp6918-bib-0063] Mossman KD , Campi G , Groves JT & Dustin ML (2005). Altered TCR signaling from geometrically repatterned immunological synapses. Science 310, 1191–1193. 16293763 10.1126/science.1119238

[tjp6918-bib-0064] Muller JM , Davis MJ , Kuo L & Chilian WM (1999). Changes in coronary endothelial cell Ca^2+^ concentration during shear stress‐ and agonist‐induced vasodilation. Am J Physiol Heart Circ Physiol 276, H1706–1714. 10.1152/ajpheart.1999.276.5.H1706 10330257

[tjp6918-bib-0065] Ohashi M , Faraci F & Heistad D (2005). Peroxynitrite hyperpolarizes smooth muscle and relaxes internal carotid artery in rabbit via ATP‐sensitive K^+^ channels. Am J Physiol Heart Circ Physiol 289, H2244–2250. 16219814 10.1152/ajpheart.00254.2005

[tjp6918-bib-0066] Olson ML , Chalmers S & McCarron JG (2010). Mitochondrial Ca^2+^ uptake increases Ca^2+^ release from inositol 1,4,5‐trisphosphate receptor clusters in smooth muscle cells. J Biol Chem 285, 2040–2050. 19889626 10.1074/jbc.M109.027094 PMC2804361

[tjp6918-bib-0067] Olson ML , Sandison ME , Chalmers S & McCarron JG (2012). Microdomains of muscarinic acetylcholine and Ins(1,4,5)P_3_ receptors create ‘Ins(1,4,5)P_3_ junctions’ and sites of Ca^2+^ wave initiation in smooth muscle. J Cell Sci 125, 5315–5328. 22946060 10.1242/jcs.105163 PMC3561854

[tjp6918-bib-0068] Paniagua OA , Bryant MB & Panza JA (2000). Transient hypertension directly impairs endothelium‐dependent vasodilation of the human microvasculature. Hypertension 36, 941–944. 11116104 10.1161/01.hyp.36.6.941

[tjp6918-bib-0069] Patel AJ , Lazdunski M & Honore E (2001). Lipid and mechano‐gated 2P domain K^+^ channels. Curr Opin Cell Biol 13, 422–428. 11454447 10.1016/s0955-0674(00)00231-3

[tjp6918-bib-0070] Phillips SA , Das E , Wang J , Pritchard K & Gutterman DD (2011). Resistance and aerobic exercise protects against acute endothelial impairment induced by a single exposure to hypertension during exertion. J Appl Physiol 110, 1013–1020. 21252216 10.1152/japplphysiol.00438.2010 PMC3075126

[tjp6918-bib-0071] Plane F , Wiley KE , Jeremy JY , Cohen RA & Garland CJ (1998). Evidence that different mechanisms underlie smooth muscle relaxation to nitric oxide and nitric oxide donors in the rabbit isolated carotid artery. Br J Pharmacol 123, 1351–1358. 9579730 10.1038/sj.bjp.0701746 PMC1565301

[tjp6918-bib-0072] Popp R , Fleming I & Busse R (1998). Pulsatile stretch in coronary arteries elicits release of endothelium‐derived hyperpolarizing factor: a modulator of arterial compliance. Circ Res 82, 696–703. 9546378 10.1161/01.res.82.6.696

[tjp6918-bib-0073] Qi H , Li L & Shuai J (2015). Optimal microdomain crosstalk between endoplasmic reticulum and mitochondria for Ca^2+^ oscillations. Sci Rep 5, 7984. 25614067 10.1038/srep07984 PMC4303883

[tjp6918-bib-0074] Qian X , Francis M , Solodushko V , Earley S & Taylor MS (2013). Recruitment of dynamic endothelial Ca^2+^ signals by the TRPA1 channel activator AITC in rat cerebral arteries. Microcirculation 20, 138–148. 22928941 10.1111/micc.12004 PMC3524345

[tjp6918-bib-0075] Ranade SS , Qiu Z , Woo SH , Hur SS , Murthy SE , Cahalan SM , Xu J , Mathur J , Bandell M , Coste B , Li YS , Chien S & Patapoutian A (2014). Piezo1, a mechanically activated ion channel, is required for vascular development in mice. Proc Natl Acad Sci USA 111, 10347–10352. 24958852 10.1073/pnas.1409233111 PMC4104881

[tjp6918-bib-0076] Rizzuto R , Pinton P , Carrington W , Fay FS , Fogarty KE , Lifshitz LM , Tuft RA & Pozzan T (1998). Close contacts with the endoplasmic reticulum as determinants of mitochondrial Ca^2+^ responses. Science 280, 1763–1766. 9624056 10.1126/science.280.5370.1763

[tjp6918-bib-0077] Saunter CD , Semprini S , Buckley C , Mullins J & Girkin JM (2012). Micro‐endoscope for in vivo widefield high spatial resolution fluorescent imaging. Biomed Opt Express 3, 1274–1278. 22741074 10.1364/BOE.3.001274 PMC3370968

[tjp6918-bib-0078] Schindelin J , Arganda‐Carreras I , Frise E , Kaynig V , Longair M , Pietzsch T , Preibisch S , Rueden C , Saalfeld S , Schmid B , Tinevez JY , White DJ , Hartenstein V , Eliceiri K , Tomancak P & Cardona A (2012). Fiji: an open‐source platform for biological‐image analysis. Nat Methods 9, 676–682. 22743772 10.1038/nmeth.2019 PMC3855844

[tjp6918-bib-0079] Sheetz MP (1993). Glycoprotein motility and dynamic domains in fluid plasma membranes. Annu Rev Biophys Biomol Struct 22, 417–431. 8347996 10.1146/annurev.bb.22.060193.002221

[tjp6918-bib-0080] Shiu YT , Li S , Marganski WA , Usami S , Schwartz MA , Wang YL , Dembo M & Chien S (2004). Rho mediates the shear‐enhancement of endothelial cell migration and traction force generation. Biophys J 86, 2558–2565. 15041692 10.1016/S0006-3495(04)74311-8 PMC1304103

[tjp6918-bib-0081] Smith GD (1996). Analytical steady‐state solution to the rapid buffering approximation near an open Ca^2+^ channel. Biophys J 71, 3064–3072. 8968577 10.1016/S0006-3495(96)79500-0 PMC1233795

[tjp6918-bib-0082] Socha MJ , Behringer EJ & Segal SS (2012 *a*). Calcium and electrical signalling along endothelium of the resistance vasculature. Basic Clin Pharmacol Toxicol 110, 80–86. 21917120 10.1111/j.1742-7843.2011.00798.x PMC3271116

[tjp6918-bib-0083] Socha MJ , Domeier TL , Behringer EJ & Segal SS (2012 *b*). Coordination of intercellular Ca^2+^ signaling in endothelial cell tubes of mouse resistance arteries. Microcirculation 19, 757–770. 22860994 10.1111/micc.12000 PMC3502682

[tjp6918-bib-0084] Sonkusare SK , Bonev AD , Ledoux J , Liedtke W , Kotlikoff MI , Heppner TJ , Hill‐Eubanks DC & Nelson MT (2012). Elementary Ca^2+^ signals through endothelial TRPV4 channels regulate vascular function. Science 336, 597–601. 22556255 10.1126/science.1216283 PMC3715993

[tjp6918-bib-0085] Spassova MA , Hewavitharana T , Xu W , Soboloff J & Gill DL (2006). A common mechanism underlies stretch activation and receptor activation of TRPC6 channels. Proc Natl Acad Sci USA 103, 16586–16591. 17056714 10.1073/pnas.0606894103 PMC1637625

[tjp6918-bib-0086] Strotmann R , Harteneck C , Nunnenmacher K , Schultz G & Plant TD (2000). OTRPC4, a nonselective cation channel that confers sensitivity to extracellular osmolarity. Nat Cell Biol 2, 695–702. 11025659 10.1038/35036318

[tjp6918-bib-0087] Strotmann R , Schultz G & Plant TD (2003). Ca^2+^‐dependent potentiation of the nonselective cation channel TRPV4 is mediated by a C‐terminal calmodulin binding site. J Biol Chem 278, 26541–26549. 12724311 10.1074/jbc.M302590200

[tjp6918-bib-0088] Sun D , Huang A , Recchia FA , Cui Y , Messina EJ , Koller A & Kaley G (2001). Nitric oxide‐mediated arteriolar dilation after endothelial deformation. Am J Physiol Heart Circ Physiol 280, H714–721. 11158970 10.1152/ajpheart.2001.280.2.H714

[tjp6918-bib-0089] Tanko LB , Mikkelsen EO & Simonsen U (1999). A new experimental approach in endothelium‐dependent pharmacological investigations on isolated porcine coronary arteries mounted for impedance planimetry. Br J Pharmacol 128, 165–173. 10498848 10.1038/sj.bjp.0702752 PMC1571598

[tjp6918-bib-0090] Thi MM , Tarbell JM , Weinbaum S & Spray DC (2004). The role of the glycocalyx in reorganization of the actin cytoskeleton under fluid shear stress: a “bumper‐car” model. Proc Natl Acad Sci USA 101, 16483–16488. 15545600 10.1073/pnas.0407474101 PMC534550

[tjp6918-bib-0091] Toda N , Inoue S , Okunishi H & Okamura T (1990). Intra‐ and extraluminally‐applied acetylcholine on the vascular tone or the response to transmural stimulation in dog isolated mesenteric arteries. Naunyn Schmiedebergs Arch Pharmacol 341, 30–36. 2314481 10.1007/BF00195054

[tjp6918-bib-0092] Toda N , Minami Y & Onoue H (1988). Extraluminally applied acetylcholine and oxyhemoglobin on the release and action of EDRF. Eur J Pharmacol 151, 123–126. 3262068 10.1016/0014-2999(88)90700-5

[tjp6918-bib-0093] Tran CH , Taylor MS , Plane F , Nagaraja S , Tsoukias NM , Solodushko V , Vigmond EJ , Furstenhaupt T , Brigdan M & Welsh DG (2012). Endothelial Ca^2+^ wavelets and the induction of myoendothelial feedback. Am J Physiol Cell Physiol 302, C1226–1242. 22277756 10.1152/ajpcell.00418.2011 PMC3330726

[tjp6918-bib-0094] Tu H , Wang Z , Nosyreva E , De Smedt H & Bezprozvanny I (2005). Functional characterization of mammalian inositol 1,4,5‐trisphosphate receptor isoforms. Biophys J 88, 1046–1055. 15533917 10.1529/biophysj.104.049593 PMC1305111

[tjp6918-bib-0095] Tzima E , Irani‐Tehrani M , Kiosses WB , Dejana E , Schultz DA , Engelhardt B , Cao G , DeLisser H & Schwartz MA (2005). A mechanosensory complex that mediates the endothelial cell response to fluid shear stress. Nature 437, 426–431. 16163360 10.1038/nature03952

[tjp6918-bib-0096] Vecchione C , Carnevale D , Di Pardo A , Gentile MT , Damato A , Cocozza G , Antenucci G , Mascio G , Bettarini U , Landolfi A , Iorio L , Maffei A & Lembo G (2009). Pressure‐induced vascular oxidative stress is mediated through activation of integrin‐linked kinase 1/βPIX/Rac‐1 pathway. Hypertension 54, 1028–1034. 19770407 10.1161/HYPERTENSIONAHA.109.136572

[tjp6918-bib-0097] Wallace SM , Yasmin , McEniery CM , Maki‐Petaja KM , Booth AD , Cockcroft JR & Wilkinson IB (2007). Isolated systolic hypertension is characterized by increased aortic stiffness and endothelial dysfunction. Hypertension 50, 228–233. 17502493 10.1161/HYPERTENSIONAHA.107.089391

[tjp6918-bib-0098] Yamamoto K , Sokabe T , Matsumoto T , Yoshimura K , Shibata M , Ohura N , Fukuda T , Sato T , Sekine K , Kato S , Isshiki M , Fujita T , Kobayashi M , Kawamura K , Masuda H , Kamiya A & Ando J (2006). Impaired flow‐dependent control of vascular tone and remodeling in P2X4‐deficient mice. Nat Med 12, 133–137. 16327800 10.1038/nm1338

[tjp6918-bib-0099] Yeung T , Georges PC , Flanagan LA , Marg B , Ortiz M , Funaki M , Zahir N , Ming W , Weaver V & Janmey PA (2005). Effects of substrate stiffness on cell morphology, cytoskeletal structure, and adhesion. Cell Motil Cytoskeleton 60, 24–34. 15573414 10.1002/cm.20041

[tjp6918-bib-0100] Zhao H , Pykalainen A & Lappalainen P (2011). I‐BAR domain proteins: linking actin and plasma membrane dynamics. Curr Opin Cell Biol 23, 14–21. 21093245 10.1016/j.ceb.2010.10.005

[tjp6918-bib-0101] Zhao Y , Flavahan S , Leung SW , Xu A , Vanhoutte PM & Flavahan NA (2015). Elevated pressure causes endothelial dysfunction in mouse carotid arteries by increasing local angiotensin signaling. Am J Physiol Heart Circ Physiol 308, H358–363. 25485905 10.1152/ajpheart.00775.2014 PMC4329479

[tjp6918-bib-0102] Zimmerberg J & Kozlov MM (2006). How proteins produce cellular membrane curvature. Nat Rev Mol Cell Biol 7, 9–19. 16365634 10.1038/nrm1784

